# Carrier of Wingless (Cow) Regulation of *Drosophila* Neuromuscular Junction Development

**DOI:** 10.1523/ENEURO.0285-19.2020

**Published:** 2020-03-03

**Authors:** Danielle L. Kopke, Shannon N. Leahy, Dominic J. Vita, Sofia C. Lima, Zachary L. Newman, Kendal Broadie

**Affiliations:** 1 Department of Biological Sciences, Kennedy Center for Research on Human Development, Vanderbilt University, Nashville, Tennessee 37235; 2 Department of Molecular and Cell Biology, University of California, Berkeley, Berkeley, California 94720

**Keywords:** Drosophila, HSPG, neuromuscular junction, synaptomatrix

## Abstract

The first Wnt signaling ligand discovered, *Drosophila* Wingless [Wg (Wnt1 in mammals)], plays critical roles in neuromuscular junction (NMJ) development, regulating synaptic architecture, and function. Heparan sulfate proteoglycans (HSPGs), consisting of a core protein with heparan sulfate (HS) glycosaminoglycan (GAG) chains, bind to Wg ligands to control both extracellular distribution and intercellular signaling function. *Drosophila* HSPGs previously shown to regulate Wg trans-synaptic signaling at the NMJ include the glypican Dally-like protein (Dlp) and perlecan Terribly Reduced Optic Lobes (Trol). Here, we investigate synaptogenic functions of the most recently described *Drosophila* HSPG, secreted Carrier of Wingless (Cow), which directly binds Wg in the extracellular space. At the glutamatergic NMJ, we find that Cow secreted from the presynaptic motor neuron acts to limit synaptic architecture and neurotransmission strength. In c*ow* null mutants, we find increased synaptic bouton number and elevated excitatory current amplitudes, phenocopying presynaptic Wg overexpression. We show *cow* null mutants exhibit an increased number of glutamatergic synapses and increased synaptic vesicle fusion frequency based both on GCaMP imaging and electrophysiology recording. We find that membrane-tethered Wg prevents *cow* null defects in NMJ development, indicating that Cow mediates secreted Wg signaling. It was shown previously that the secreted Wg deacylase Notum restricts Wg signaling at the NMJ, and we show here that Cow and Notum work through the same pathway to limit synaptic development. We conclude Cow acts cooperatively with Notum to coordinate neuromuscular synapse structural and functional differentiation via negative regulation of Wg trans-synaptic signaling within the extracellular synaptomatrix.

## Significance Statement

Wnt intercellular signaling is disrupted in numerous devastating neurologic disorders, including Alzheimer’s disease. Therefore, an understanding of Wnt signaling regulation is important for the design and implementation of targeted treatments. As a disease model, the *Drosophila* glutamatergic neuromuscular junction (NMJ) system is large, accessible, and genetically malleable, and thus well suited for discovering the molecular and cellular mechanisms of Wnt signaling regulation. Extracellular heparan sulfate proteoglycans (HSPGs) are important players as regulators of Wnt intercellular signaling. Here, we show secreted HSPG Carrier of Wingless (Cow), which directly binds to the founding Wnt-1 ligand, regulates NMJ structure and function. The mammalian homolog of Cow, Testican-2, is highly expressed in the brain. Studying this HSPG in *Drosophila* should inform mechanisms of Wnt regulation in human brain.

## Introduction

The developing nervous system requires the coordinated action of many signaling molecules to ensure proper synapse formation and function. One key class of signals is the Wnt ligands. The first discovered Wnt, *Drosophila* Wingless (Wg), is secreted from presynaptic neurons (Packard et al., 2002) and glia (Kerr et al., 2014) at the developing glutamatergic neuromuscular junction (NMJ) to bind to the Frizzled-2 (Fz2) receptor (Bhanot et al., 1996) in both anterograde and autocrine signaling. In the postsynaptic muscle, Wg binding to Fz2 activates the noncanonical Frizzled Nuclear Import (FNI) pathway, which leads to Fz2 endocytosis and cleavage of the Fz2 C terminus (Fz2-C; Mathew et al., 2005). The Fz2-C fragment is trafficked to the nucleus to control translation of synaptic mRNAs and glutamate receptors (GluRs; Speese et al., 2012). In presynaptic neurons, Wg binding to Fz2 activates a divergent canonical pathway inhibiting glycogen synthase kinase 3β (GSK3β) homolog Shaggy (Sgg) to control microtubule cytoskeletal dynamics via the microtubule-associated protein 1B (MAP1B) homolog Futsch (Miech et al., 2008), resulting in synaptic bouton growth (Franco et al., 2004; Ataman et al., 2008). The Wg signaling ligand must be tightly regulated in the synaptic extracellular space (synaptomatrix) to ensure proper NMJ development.

One critical category of proteins regulating Wg ligand in the synaptomatrix is heparan sulfate proteoglycans (HSPGs; Kamimura and Maeda, 2017). HSPGs consist of a core protein to which heparan sulfate (HS) glycosylphosphatidylinositol (GAG) chains are covalently attached. HS GAG chains are composed of repeating disaccharide subunits expressing variable sulfation patterns (the “sulfation code”; Masu, 2016). These GAG chains bind secreted extracellular ligands to regulate intercellular signaling. There are three HSPG families: transmembrane; glycerophosphatidylinositol (GPI) anchored; and secreted. The *Drosophila* genome encodes only five HSPGs, with the following three known to affect NMJ development: transmembrane syndecan (Johnson et al., 2006); GPI-anchored Dally-like protein (Dlp; Johnson et al., 2006; Dani et al., 2012); and secreted perlecan (Kamimura et al., 2013). A second secreted HSPG recently characterized in *Drosophila* was named Carrier of Wingless (Cow; Chang and Sun, 2014). In the developing wing disk, Cow directly binds secreted Wg and promotes its extracellular transport in an HS-dependent manner. Cow shows a biphasic effect on Wg target genes. Removing Cow results in a Wg overexpression (OE) phenotype for short-range targets, and a loss-of-function phenotype for long-range targets (Chang and Sun, 2014).

The mammalian homolog of Cow, Testican-2, is highly expressed within the developing mouse brain (Vannahme et al., 1999), and inhibits neurite extension in cultured neurons (Schnepp et al., 2005), although the mechanism of action is not known. We therefore set out to characterize Cow functions at the developing *Drosophila* NMJ. We use the larval NMJ model because it is large, accessible and particularly well characterized for HSPG-dependent Wg trans-synaptic signaling (Sears and Broadie, 2018). Each NMJ terminal consists of a relatively stereotypical innervation pattern, with consistent axonal branching and synaptic bouton formation (Menon et al., 2013). Boutons are the functional unit of the NMJ, containing presynaptic components required for neurotransmission including glutamate-containing synaptic vesicle (SV) pools and specialized active zone (AZ) sites for SV fusion. AZs contain Bruchpilot (Brp) scaffolds, which both cluster Ca^2+^ channels (Kittel et al., 2006) and tether SVs (Hallermann et al., 2010). AZs are directly apposed to GluR clusters in the postsynaptic muscle membrane (Schuster et al., 1991). This spatially precise juxtaposition is critical for high-speed and efficient synaptic communication between neuron and muscle.

In this study, we sought to test Cow functions at the NMJ, with the hypothesis that Cow should facilitate extracellular Wg transport across the synapse. Structurally, *cow* null mutants display overelaborated NMJs with more boutons and more synapses, phenocopying Wg overexpression. This phenotype is replicated with targeted neuronal Cow knockdown, but not muscle Cow knockdown, which is consistent with Cow secretion from the presynaptic terminal. Functionally, *cow* null mutants display increased synaptic transmission strength. Both electrophysiology recording and postsynaptically targeted GCaMP imaging show increased SV fusion, indicating elevated presynaptic function. Replacing native Wg with a membrane-tethered Wg blocks secretion (Alexandre et al., 2014). Tethered Wg has little effect on NMJ development, but when combined with the *cow* null suppresses the synaptic bouton increase, indicating that Cow mediates only secreted Wg signaling. It was recently shown that Notum, a secreted Wg deacylase, also restricts Wg signaling at the NMJ (Kopke et al., 2017). We show here that combining null *cow* and *notum* heterozygous mutants causes a synergistic increase in NMJ development, indicating nonallelic noncomplementation. Moreover, combining null *cow* and *notum* homozygous mutants did not cause an increase in NMJ development compared with the single nulls, indicating an interaction within the same pathway. We conclude that Cow functions via negative regulation of Wg trans-synaptic signaling.

## Materials and Methods

### *Drosophila* genetics

All *Drosophila* stocks were reared on standard cornmeal/agar/molasses food at 25°C in a 12 h light/dark cycling incubator. Mixed sexes were used for all experiments except the SynapGCaMP imaging (females only). The genetic background control was *w^1118^*. The *cow^5Δ^* mutant, *UAS-cow-miRNA-1* (referred to as *UAS-cow-RNAi*) and *UAS-SP-eGFP-cow* (referred to as *UAS-Cow::eGFP*) lines (Chang and Sun, 2014) were obtained from Yi Henry Sun (Institute of Molecular Biology, Academia Sinica, Taipei, Taiwan). The *cow^GDP^* #03259 (y[1] w[*]; Mi{y[+mDint2]=MIC}Cow[MI03259]/TM3, Sb[1] Ser[1]) and *cow^GDP^* #12802 (y[1] w[*]; Mi{y[+mDint2]=MIC}Cow[MI12802]) mutants, and the *cow* Df #6193 (w[1118]; Df(3R)Exel6193, P{w[+mC]=XP-U}Exel6193/TM6B, Tb[1]) and *cow* Df #619 (w[1118]; Df(3R)BSC619/TM6C, cu[1] Sb[1]) deficiencies were all obtained from the Bloomington *Drosophila* Stock Center (stock #40757, #58669, 7672, and 25694, respectively; Indiana University, Bloomington, IN). Cow-Gal4 was obtained from the Vienna Tile (VT) collection of the Vienna *Drosophila* Resource Center (VT046086; Vienna, Austria). Neuronal *vesicular glutamate transporter* (*vglut*)*-Gal4* and muscle-specific *24B-Gal4* driver lines were obtained from the Bloomington *Drosophila* Stock Center. The *MHC-CD8-GCaMP6f-Sh* Ca^2+^ reporter (SynapGCaMP6f; Newman et al., 2017) was obtained from Ehud Isacoff (University of California, Berkeley, CA). Control *wg{KO; FRT Wg FRT QF; pax-Cherry}* and membrane-tethered *wg{KO; FRT NRT-Wg FRT QF; pax-Cherry}* (Alexandre et al., 2014) were obtained with permission from Andrea Page-McCaw (Department of Cell and Developmental Biology, Vanderbilt University, Nashville, TN). Null *notum^KO^ (4)(w+)* (Kakugawa et al., 2015) was obtained from Jean-Paul Vincent (Francis Crick Institute, London, UK).

### PCR/RT-PCR studies

Staged *Drosophila* eggs were dechorionated using bleach for 30 s, washed with distilled H_2_O (dH_2_O) three times, and embryos were genotyped using a GFP marker with an epifluorescent microscope. Five embryos per genotype were homogenized in 10 μl of Gloor and Engels DNA extraction buffer (10 mm Tris HCL, pH 8.2; 1 mm EDTA, pH 8.0; 25 mm NaCl; and 200 μg/ml Proteinase K) with a glass rod in an Eppendorf tube, and the homogenate was incubated at 37°C for 30 min, and then at 95°C for 2 min. For each PCR, ∼10 ng of DNA was used with the following primers: forward 5′-GCAACATTCTGGCTTCGTGTCATGC-3′ and reverse 5′-CTCTCGACTTGCAAATAGCAGACGATGATC-3′ for the *cow* gene (product size, 1927); and forward 5′-GTGGAAAAGCGGTTGAAATAGGG-3′ and reverse 5′-GTCCACATCCACAAAGATGCC-3′ for the *dfmr1* gene control (product size, 3850). For the RT-PCR studies, one embryo per genotype was used with the RNeasy Micro Kit (catalog #74004, Qiagen) to extract RNA. The OneStep RT-PCR Kit (catalog #210212, Qiagen) was used. For each reaction, ∼7 ng of RNA was used with the following primers: forward 5′-AGAACAGCAACTTGAATGCCTATC-3′ and reverse 5′-CGAAGCATCTGCACCATTCC-3′ for the *cow* gene (product size, 348); and forward 5′-TAAACTGCGAGAGGTTTTCC-3′ and reverse 5’ ATTCGATGAGTGTACGCTG-3′ for the *dmgalectin* gene control (product size, 321). Products were loaded on a 0.8% agarose gel in TAE buffer with purple gel loading dye (catalog #B7025S, New England Biolabs) and SYBR safe DNA gel stain (catalog #S33102, Thermo Fisher Scientific), and run at 100 V for 30 min.

### Cow antibodies

**Figure 1.**
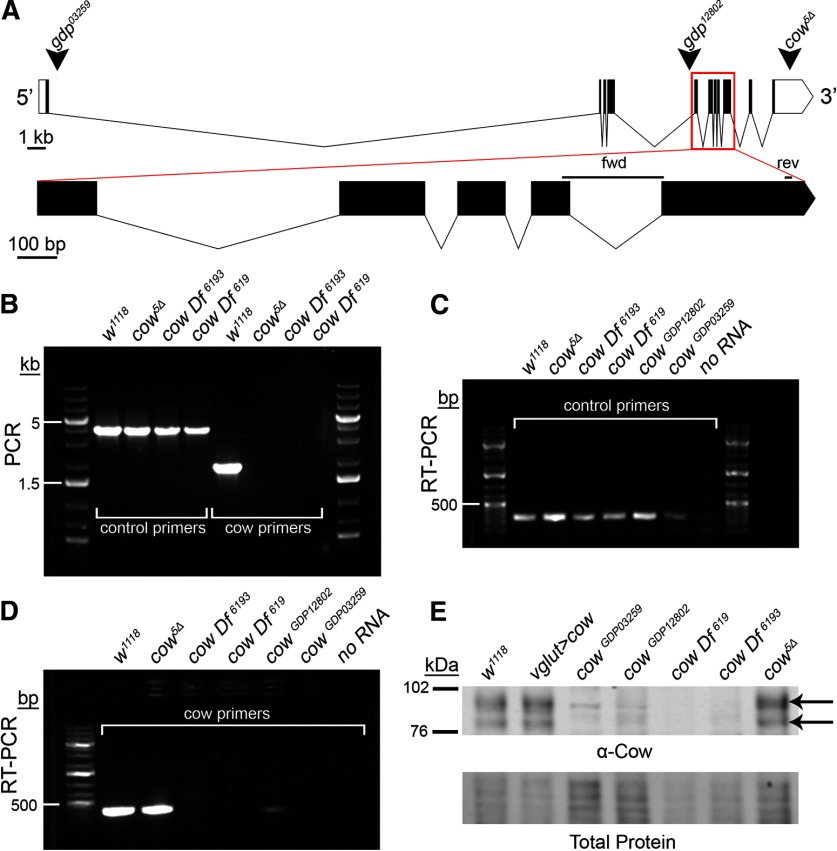
Carrier of Wingless (Cow) genetic locus and mutant characterization. ***A***, Intron/exon structure of the *cow* gene (transcript *cow-RD*; flybase.org). Arrowheads indicate gene disruption project (gdp) inserts in two different lines (03259 and 12802). The third arrowhead indicates where the published *cow^5Δ^* deletion begins in the 3′ UTR and runs 9119 bp downstream (Chang and Sun, 2014). Below, the expanded region outlined with the red box is labeled “fwd” and “rev” to depict the RT-PCR primer pair. ***B***, PCR products from the genotypes listed. Control (*dfmr1* gene) and *cow* primers from the region of the *cow^5Δ^* deletion. ***C***, ***D***, RT-PCR products from the genotypes listed using both control (*dmgalectin* gene) and *cow* primers. ***E***, Western blot of the indicated genotypes using an anti-Cow antibody, with the total protein stain shown below. The two arrows indicate Cow protein with and without GAG chains.

**Figure 2.**
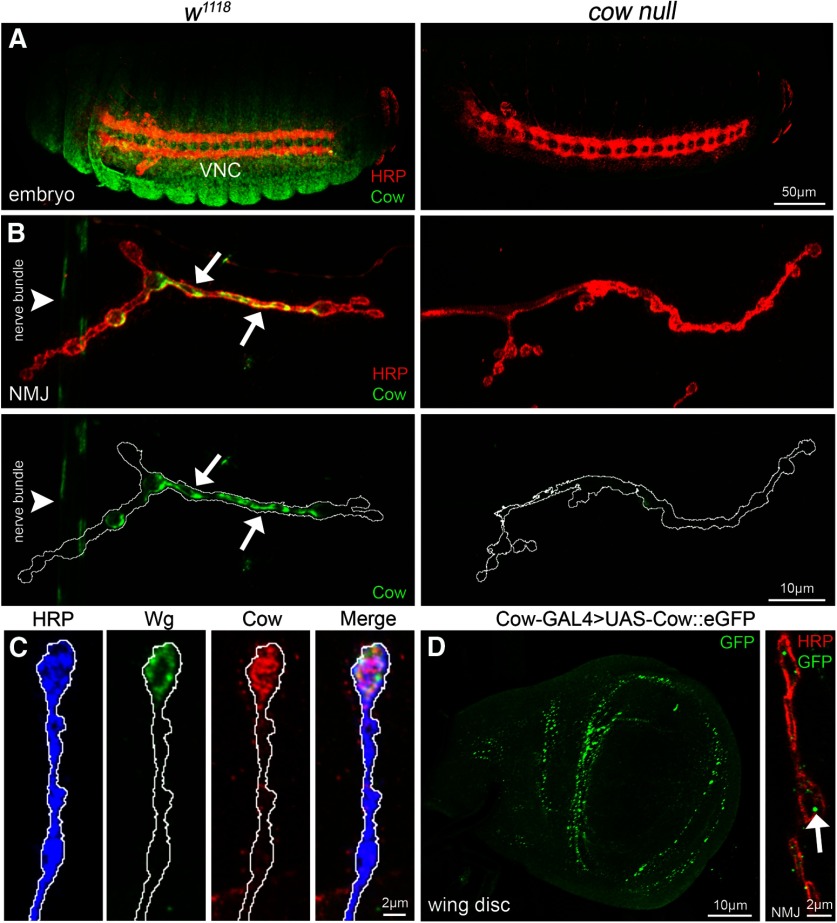
Cow expression in embryos, larval NMJ synaptic terminal, and wing disk. ***A***, Confocal images of stage 16 embryos colabeled with anti-HRP (red) to mark neuronal membranes and anti-Cow (green) in genetic background control (*w^1118^*, left) and *cow* null (*cow^GDP^/cow^GDP^*, right). The ventral nerve cord (VNC) is labeled. ***B***, Confocal images of third instar NMJ colabeled with anti-HRP (red) and anti-Cow (green) in control (*w^1118^*, left) and *cow* null (*cow^GDP^/cow^GDP^*, right). From nonpermeabilized labeling, Cow appears secreted from a dynamic subset of synaptic boutons (arrows) and also present in the nerve bundle (arrowhead). Cow is shown without HRP in below images. White line marks the NMJ terminal HRP domain. ***C***, Higher-magnification images of *w^1118^*NMJ synaptic boutons colabeled with anti-HRP (blue), anti-Wg (green), and anti-Cow (red), with merged image on right. White line marks the NMJ terminal HRP domain. ***D***, Cow-GAL4 driving UAS-Cow::eGFP in wandering third instar wing imaginal disk (left) and NMJ colabeled with anti-HRP (red) and anti-GFP (green, right). For the NMJ, a single confocal section (0.5 μm) shows Cow punctae (arrow) within and surrounding synaptic boutons.

We used a well characterized, published anti-Cow antibody (Chang and Sun, 2014). New rabbit anti-Cow antibodies were also made by ABclonal against amino acids 36–236. Three antiserums were recovered and affinity purified (29, 30, 31). Cow antibody 31 was preabsorbed against *cow* nulls (*cow^GDP^*) for imaging studies. Cow antibody 31 was used for Figures 1, 2 and 4).

### Western blotting

Staged *Drosophila* eggs (18–24 h postfertilization for maximum expression; www.fruitfly.org) were dechorionated using bleach for 30 s and washed with dH_2_O three times, and embryos were genotyped using a GFP marker with an epifluorescent microscope. Twenty-five embryos were placed into an Eppendorf tube with 24 μl RIPA buffer (150 mm sodium chloride, 1% Triton X-100, 0.5% sodium deoxycholate, 0.1% sodium dodecyl sulfate, 50 mm Tris) and protease inhibitor cocktail (catalog #P8340, Sigma-Aldrich), then immediately snap frozen in a dry ice ethanol bath. Samples were sonicated (settings: 90% duty, output 2; Sonifier 250, Branson) for 20 s, vortexed (speed 4; Standard Mini Vortexer, VMR Scientific Products) for 5 s, and then centrifuged at 14,000 rpm for 10 min. The supernatant was then transferred to new tubes with 1× Invitrogen NuPage LDS buffer (catalog #NP007, Thermo Fisher Scientific) and 5% 2-mercaptoethanol (catalog #M7154, Sigma-Aldrich), then vortexed as above. Samples were incubated at room temperature for 20 min, heated at 100°C for 10 min, then centrifuged as above. Equal volumes of lysate were loaded into precast NuPage 4–12% Invitrogen Bis-Tris gels (catalog #NP0336, Thermo Fisher Scientific) with Life Technologies NuPage running buffer (catalog #NP002, Thermo Fisher Scientific) and Invitrogen NuPage antioxidant (catalog #NP0005, Thermo Fisher Scientific). Electrophoresis was performed at 150 V for 2 h. Protein was then transferred overnight at 4°C with constant 30 mA current to nitrocellulose membranes (catalog #Protran NBA085C001EA, PerkinElmer) in the Life Technologies NuPage transfer buffer (catalog #NP0006-1, Thermo Fisher Scientific) supplemented with 20% methanol (catalog # AH230-4, Honeywell). Following transfer, membranes were rinsed with dH_2_O, air dried at room temperature for 1 h, and then blocked with 2% nonfat powdered milk in TBS-T (0.1% Tween-20, 150 mm sodium chloride, 5 mm potassium chloride, and 25 mm Tris, pH 7.6) at room temperature for 1 h with rotation. Primary antibodies were incubated overnight in 2% milk in TBST. Membranes were then washed in TBST (five times for 6 min), followed by incubation in secondary antibody at room temperature in 2% milk in TBST for 1 h with rotation, and washed again as before. Imaging was performed on a LI-COR Odyssey Imager with analysis on Image Studio Lite (LI-COR). Total protein was assessed via the REVERT total protein stain (catalog #926–11 011, LI-COR). Primary antibodies: rabbit anti-Cow (Ab 31, this study) and goat anti-GFP (catalog #ab6662, Abcam), both at 1:1000. The following secondary antibodies were used: IRDye 680 donkey anti-rabbit (catalog #926–68 073, LI-COR) and IRDye 800 donkey anti-goat (catalog #926–32 214, LI-COR), both at 1:10,000.

### Confocal imaging

Wandering third instars were dissected in physiological solution containing the following (in mm): 128 NaCl, 2 KCl, 0.2 CaCl_2_, 4 MgCl_2_, 70 sucrose, 5 HEPES {2-[4-(2-hydroxyethyl)piperazin-1-yl]ethanesulfonic acid} at pH 7.2. The samples were fixed with 4% paraformaldehyde (catalog #15 714, EMS) diluted in PBS (catalog #46–013-CM, Corning). For intracellular labeling, samples were permeabilized with 0.2% Triton X-100 (catalog #BP151-100, Thermo Fisher Scientific) three times for 10 min each. Embryos were bleached for dechorionation, fixed with heptane and paraformaldehyde, and devitillinized with methanol. The following primary antibodies were used: mouse anti-Discs Large (DLG; 1:250; catalog #4F3), mouse anti-Wg (1:1; catalog #4D4), and mouse anti-Brp (1:200, catalog #nc82), all from the Developmental Studies Hybridoma Bank; Alexa Fluor 488-conjugated goat anti-horseradish peroxidase (HRP; 1:250; catalog #123–545-021), Cy3-conjugated goat anti-HRP (1:250; catalog #123–165-021), and Alexa Fluor 647-conjugated goat anti-HRP (catalog #123–605-021; 1:250), all from Jackson ImmunoResearch; rabbit anti-GluRIIC (1:5000; Marrus et al., 2004); and rabbit anti-GFP (1:1500; catalog #ab290, abcam). Preparations were incubated with primary antibodies overnight at 4°C and secondary antibodies at room temperature for 2 h, washed three times for 10 min each, and then mounted in Fluoromount-G (catalog #17984–25, EMS) onto 25 × 75 × 1 mm slides (Fisher Scientific, 12–544-2) with a 22 × 22-1 coverslip (catalog #12–542-B, Thermo Fisher Scientific) and sealed with clear nail polish (Sally Hansen). Imaging was performed on a Zeiss LSM 510 META laser-scanning confocal microscope, with images projected in Zen (Zeiss) and analyzed using ImageJ (NIH). NMJ intensity measurements were made with HRP signal-delineated *z*-stack areas of maximum projection using ImageJ threshold and wand-tracing tools.

### Two-electrode voltage-clamp electrophysiology

Wandering third instars were dissected longitudinally along the dorsal midline, internal organs were removed, and body walls were glued down (Vetbond, 3M). Peripheral motor nerves were cut at the base of the ventral nerve cord (VNC). Dissections and two-electrode voltage-clamp (TEVC) recordings were both conducted at 18°C in physiological saline as follows (in mm): 128 NaCl, 2 KCl, 4 MgCl2, 1.5 CaCl2, 70 sucrose, and 5 HEPES, pH 7.2. Preparations were imaged using a Zeiss Axioskop microscope with a Zeiss 40× water-immersion objective. Muscle 6 in abdominal segments 3–4 was impaled with two intracellular electrodes (1 mm outer diameter borosilicate capillaries; catalog #1B100F-4, World Precision Instruments) of ∼15 MΩ resistance filled with 3 m KCl. The muscles were clamped at −60 mV using an Axoclamp-2B amplifier (Axon Instruments). Spontaneous miniature excitatory junction current (mEJC) recordings were made in continuous 2 min sessions and low-pass filtered. For EJC records, the motor nerve was stimulated with a fire-polished suction electrode using 0.5 ms suprathreshold voltage stimuli at 0.2 Hz from a Grass S88 stimulator. Nerve stimulation-evoked EJC recordings were filtered at 2 kHz. To quantify EJC amplitude, 10 consecutive traces were averaged, and the average peak value was recorded. Clampex 9.0 was used for data acquisition, and Clampfit 9 was used for data analysis (Axon Instruments).

### SynapGCaMP imaging

For SynapGCaMP quantal imaging experiments, wandering third instars were dissected and type 1b NMJs were imaged in physiological saline as follows (in mm): 70 NaCl, 5 KCl, 1.5 CaCl_2_, 25 MgCl_2_, 10 NaHCO_3_, 5 trehalose, 115 sucrose, and 5 HEPES, pH 7.2. Fluorescence images were acquired with a Vivo Spinning Disk Confocal microscope (3i Intelligent Imaging Innovations), with a 63× 1.0 numerical aperture (NA) water-immersion objective (Zeiss), LaserStack 488 nm (50 mW) laser, Yokogawa CSU-X1 A1 spinning disk, and EMCCD camera (Photometrics Evolve). Image capture and analysis were performed as reported previously (Newman et al., 2017). Briefly, spontaneous events were imaged at 20 Hz (50 ms exposures, in streaming capture mode) for 30 s. Movies 1, 2 were then filtered, registered, and bleach corrected prior to Δ*F* conversion. Using the δ Δ*F* data, an XYT local maxima algorithm was applied to the thresholded Δ*F* data to identify where and when quantal release events occur (Newman et al., 2017). Quantal coordinates were used to calculate Δ*F*/*F* amplitudes and frequencies (normalized to the baseline SynapGCaMP6f 2D area).

Movie 1. SynapGCaMP imaging of spontaneous quantal events in the control NMJ. Example of muscle 4 type 1b NMJ imaged in the control (*vglut*-Gal4/+; SynapGCaMP6f/+) with quantified data shown in Figure 4. 10.1523/ENEURO.0285-19.2020.video.1

Movie 2. SynapGCaMP imaging of spontaneous quantal events in *cow* KD NMJ. Example of muscle 4 type 1b NMJ imaged following motor neuron-targeted *cow* RNAi (*vglut*-Gal4>UAS-*cow*-RNAi; SynapGCaMP6f/+) with quantified data shown in Figure 4. 10.1523/ENEURO.0285-19.2020.video.2

### Structured illumination microscopy

Dissected wandering third instar preparations were imaged using a Nikon N-SIM in 3D SIM (structured illumination microscopy) mode, configured with a 100× EX V-R diffraction grating, automated TiE inverted fluorescence microscope stand, 100× SR Apo 1.49 NA objective, Andor DU-897 EM-CCD, and 488/561 nm lasers. Image acquisition was managed through NIS-Elements (Nikon Instruments), and stacks were acquired with a 0.12 μm step size. Stack reconstruction of the raw data were used prior to rendering and analysis. To acquire larger fields of view and capture whole NMJs, SIM images were stitched together using the automated tiling method within NIS-Elements software.

### Laser-scanning confocal imaging analysis

We used Imaris Version 9.3.0 to quantify LSM (laser-scanning confocal imaging) images using the “surfaces” function to identify the number and volume of Brp punctae, as follows:

1. Open image file and click “add new surfaces” to start the wizard.
2. Algorithm settings click “segment only a region of interest” (ROI).
3. Select ROI in X, Y, and Z.
4. Select “source channel” and thresholding conditions.
5. Adjust threshold until all spots are selected.
6. Enable “split touching objects” with seed points diameter (0.4 μm).
7. Use “quality filter” to adjust selections with minimal background.
8. Click “finish” to execute all creation steps and exit the wizard.
9. Click “edit” tab and delete extraneous spots by hand.
10. Click “statistics” tab and export values to Microsoft Excel.

### SIM image analysis

We used Imaris Version 9.3.0 to quantify SIM images using the “spots” function to identify the number of Brp punctae and GluR clusters, as follows:

1. Open image file and click “add new spots” to start the wizard.
2. Algorithm settings click “segment only a region of interest” with “different spot sizes (region growing).”
3. Select ROI in X, Y, and Z.
4. Select “source channel” and click “background subtraction.”
5. Classify spots with a “quality” filter type and adjust by eye.
6. Spot regions click “local contrast.”
7. Region threshold with diameter from “region volume.”
8. Click “finish” to execute all creation steps and exit the wizard.
9. Click “edit” tab and delete extraneous spots by hand.
10. Click “statistics” tab and export values to Microsoft Excel.

### Statistical analyses

All statistical measurements were performed within GraphPad Prism (version 7.04 for Windows). The D’Agostino–Pearson K-squared normality test was performed on all datasets to check for normality. For comparisons of two genotypes, a *t* test (normally distributed) or Mann–Whitney test (not normally distributed) was performed. For all other comparisons of more than two genotypes, an ordinary one-way ANOVA (normally distributed) or Kruskal–Wallis test (not normally distributed) was performed. All graphs were made in Prism, and the data are represented in scatter plots with the mean ± SEM.

## Results

### Carrier of wingless (cow) genetic locus, mutants and expression profiles

The *cow* gene encodes three transcripts (*cow*-RC, *cow*-RD, *cow*-RE), with *cow*-RD containing a long 3′-UTR (Fig. 1*A*). We acquired a reported *cow* null mutant (*cow^5Δ^*; Chang and Sun, 2014), two mutations from the Gene Disruption Project (*cow^GDP^* 03259 and 12802; Bellen et al., 2004; Nagarkar-Jaiswal et al., 2015), and two *cow* deficiencies from the Bloomington *Drosophila* Stock Center (Df[619] and Df[6193]). The *cow^5Δ^* mutant has a 9119 bp deletion starting in the 3′-UTR that does not remove *cow* coding sequence, but is published as a well characterized protein null (Chang and Sun, 2014). The *cow^GDP^*lines are minos-mediated integration cassette (Mi{MIC}) insertions; 03259 in *cow* intron 1, and 12802 in *cow* intron 4. Df[619] completely removes *cow* and 31 other genes, while *cow* Df[6193] removes *cow* and 41 other genes. PCR tests were performed using primers in the *cow^5Δ^* deletion region (Fig. 1*A*). As expected, there are no PCR products from *cow^5Δ^* or either *cow* Df (Fig. 1*B*). Next, RT-PCR tests were performed using primers spanning an exon–exon junction to ensure mRNA amplification (Fig. 1*A*). The RNA extraction was confirmed using primers for a control gene (*dfmr1*; Fig. 1*C*). The *cow* transcript in the genetic background control *w^1118^* is present at similar levels in the *cow^5Δ^* line (Fig. 1*D*). There is no detectable *cow* transcript in either of the *cow* Dfs, or in one of the *cow^gdp^* lines (03259), and only a very faint product in the other *cow^gdp^* line (12802; Fig. 1*D*). Thus, *cow^gdp^*03259 is an RNA null allele.

The published *cow^5Δ^*mutation has been reported to have transcript levels similar to those of wild type, but to have no detectable Cow protein expression (Chang and Sun, 2014). We therefore next examined protein levels via Western blotting using the published, well characterized Cow antibody (Chang and Sun, 2014), as well as three new antibodies made for this study (see Materials and Methods). Cow protein has a predicted molecular weight of ∼75 kDa (without HS chains) and ∼100 kDA (with HS chains). The two Cow protein bands are clearly present in the *w^1118^* controls and absent in both *cow* deficiency lines (Fig. 1*E*). Cow protein is also undetectable in the *cow^gdp^* lines, even at heightened levels of protein loading (Fig. 1*E*). In stark contrast to previously published work (Chang and Sun, 2014), both Cow protein bands are present at normal levels in *cow^5Δ^* mutants (Fig. 1*E*, arrows). In our studies, *cow^5Δ^* mutants typically die as early-stage larvae, and the few escapers can be raised to the third instar only with constant care. In contrast, both *cow^gdp^* protein nulls are fully adult viable, both as homozygotes and as heterozygotes over Df[619]. Thus, our evidence indicates that *cow^5Δ^* does not affect Cow expression, but has a second site larval lethal mutation. Further, the Cow protein is not required for full adult viability. For the remainder of experiments, *cow^gdp^* 03259 and *cow* Df[619] were used, as both show complete removal of Cow RNA and protein.

To assess Cow protein expression in controls and null mutants, we performed anti-Cow labeling and Cow-Gal4 to drive UAS-Cow::eGFP (Fig. 2). In control embryos, Cow is widely expressed, including localization in the VNC (Fig. 2*A*). In *cow* null mutants (*cow^GDP^/cow^GDP^*), antibody labeling is undetectable (Fig. 2*A*, right). Since Cow has a signal peptide, and has been previously established to be secreted (Chang and Sun, 2014), we tested Cow expression at the NMJ using antibody labeling with nonpermeabilizing conditions. In the *w^1118^*control wandering third instar NMJ, Cow appears secreted from a dynamic subset of type 1b synaptic boutons (Fig. 2*B*, arrows). Cow is also present in a punctate pattern along the peripheral nerve bundle (arrowhead). In *cow* nulls, neuronal and synaptic antibody labeling is lost (Fig. 2*B*, right). Within NMJ synaptic boutons colabeled for both Cow and Wg antibody, the two secreted proteins have overlapping expression patterns, colocalizing in the extracellular synaptomatrix surrounding the same boutons (Fig. 2*C*). Using Cow-Gal4 to drive a UAS-Cow::eGFP, GFP is present throughout the wandering third instar wing imaginal disk, including punctae surrounding the wing pouch (Fig. 2*D*, left). Cow::eGFP is also present at the NMJ in punctae within and surrounding the synaptic boutons within a single confocal slice (Fig. 2*D*, right). Overall, Cow is expressed in both neuronal and non-neuronal tissue in embryos, larvae, and imaginal discs, and colocalizes with Wg at the NMJ.

### Presynaptic cow restricts NMJ growth and synaptic bouton formation

**Figure 3.**
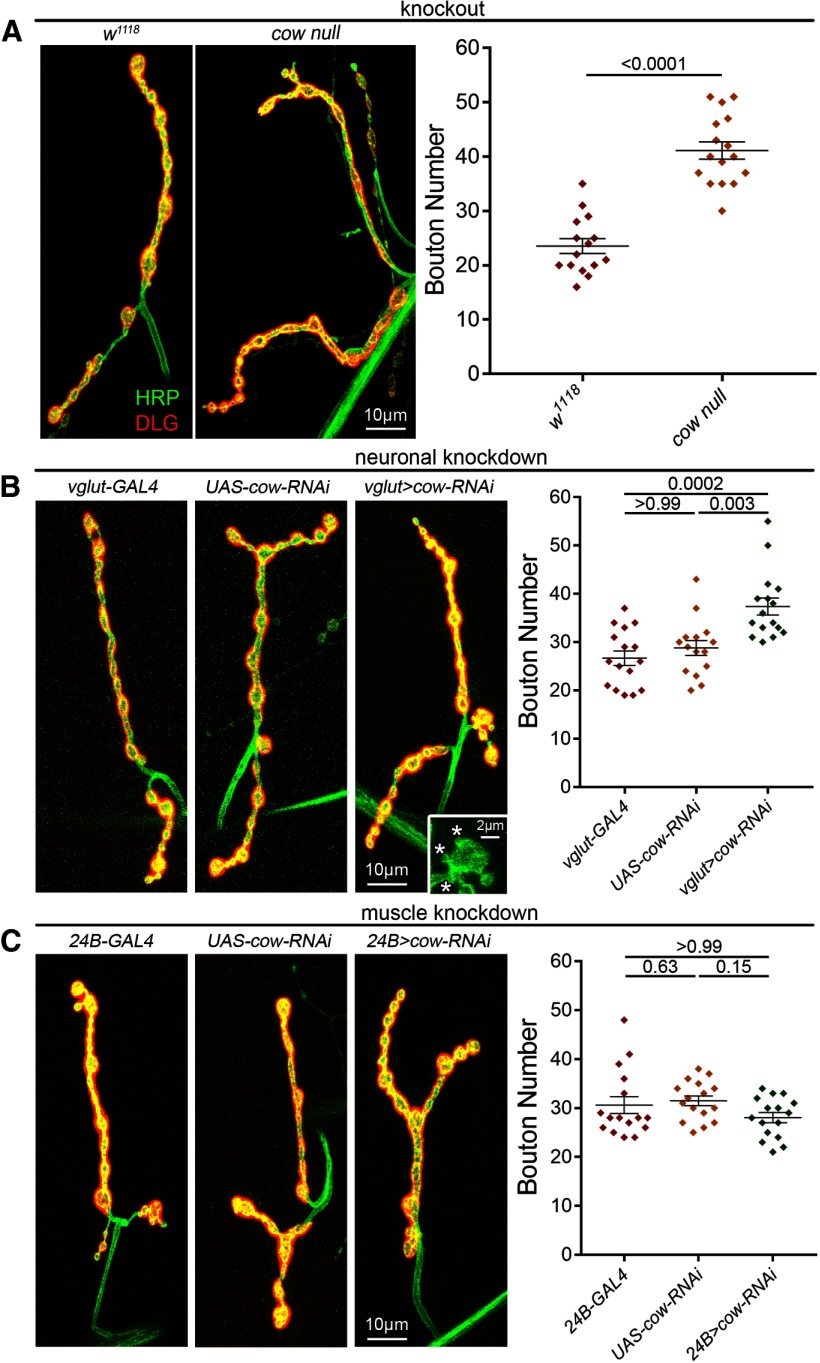
Presynaptically secreted Cow limits NMJ synaptic bouton number. ***A***, Confocal images of the muscle 4 NMJ colabeled with anti-HRP (green) to mark the presynaptic membrane and anti-DLG (red) to mark the postsynaptic domain in the genetic background control (*w^1118^*, left) and the *cow* null mutant (*cow^GDP^*/*Df*, right). Synaptic bouton number is shown in a scatter plot, with mean ± SEM. ***B***, Representative confocal NMJ images of motor neuron-targeted Gal4 driver control (*vglut*-Gal4/+; left), UAS-RNAi transgene control (UAS-*cow*-RNAi/+, middle) and *cow* RNAi knockdown (vglut>*cow*-RNAi, right). Satellite boutons (asterisks) are shown in the inset. Right, Synaptic bouton number is shown in a scatter plot, with mean ± SEM. ***C***, Representative confocal NMJ images of muscle-targeted Gal4 driver control (24B-Gal4/+, left), UAS-RNAi transgene control (UAS-*cow*-RNAi/+, middle) and *cow* RNAi knockdown (24B>*cow*-RNAi, right). Synaptic bouton number is quantified to the right. The *p* values are shown for each statistical comparison.

Wg trans-synaptic signaling regulates NMJ growth and synaptic bouton formation (Packard et al., 2002), thus we hypothesized that if Cow regulates Wg at the NMJ, Cow loss should affect the NMJ architecture. Each NMJ terminal consists of a relatively stereotypical muscle innervation pattern, with a consistent number of axon branches and large synaptic boutons (Menon et al., 2013). Wg signaling bidirectionally regulates synaptic development, with *Wg* knockdown decreasing NMJ synaptic bouton number and *Wg* OE increasing boutons (Packard et al., 2002; Kopke et al., 2017), including an increase in satellite boutons [small boutons connected to the mature (parent) bouton or adjacent axon; Torroja et al., 1999; Gatto and Broadie, 2008]. To test Cow requirements in synaptic architectural development, we labeled the wandering third instar NMJ. Anti-HRP was used to label the NMJ terminal by binding to extracellular fucosylated *N*-glycans associated with the presynaptic neural membrane (Jan and Jan, 1982; Parkinson et al., 2013). Anti-DLG was used to label the postsynaptic scaffold in the subsynaptic reticulum (SSR; Lahey et al., 1994; Parnas et al., 2001). We used *cow^GDP^/Df* (referred to as *cow* null) to eliminate *cow* globally, and characterized *cow* RNAi lines (Chang and Sun, 2014) for both motor neuron (*vglut*-Gal4) and muscle (*24B*-Gal4) cell-targeted knock-down studies. Sample images and the summary of results are shown in Figure 3.

Cow restrains NMJ development, specifically restricting synaptic bouton formation. When Cow is knocked out completely, there is a clear increase in boutons (Fig. 3*A*, left). In quantified measurements, *cow* nulls show a very significant increase in synaptic bouton number (*w^1118^*, 25.53 ± 1.37 vs *cow^GDP^/Df*, 41.13 ± 1.6*; p* < 0.0001; Fig. 3*A*, right). With targeted *cow* knockdown in presynaptic motor neurons (*vglut-*Gal4*>cow-RNAi*), there is the same increase in NMJ bouton formation (Fig. 3*B*), indicating that Cow originates from the neuron. Interestingly, presynaptic Cow knockdown also increases the number of satellite boutons (Fig. 3*B*; inset). Presynaptic *cow* knockdown causes very significantly elevated mature bouton numbers (*vglut*-Gal4*/+*, 26.69 *±* 1.49 vs *vglut>cow-RNAi*, 37.38 ± 1.75; *p* = 0.0002) as well as an increased percentage of satellite boutons (*vglut*-Gal4*/+*, 2.9 *±* 0.89% vs *vglut>cow-RNAi*, 5.77 *±* 1.86; *p* = 0.061; Fig. 3*B*, right). Conversely, postsynaptic *cow* knockdown (*24B-*Gal4>*cow-RNAi*) causes no discernable differences from the controls (Fig. 3*C*, right). Mature and satellite bouton quantifications demonstrate no effect of removing Cow from the muscle (mature; *24B/+*, 30.63 ± 1.73 vs *24B>cow-RNAi*, 28.06 ± 1.04; *p* > 0.9999; Fig. 3*C*, right; Table 1, satellite results). Together, these results show Cow originating from the presynaptic motor neuron restricts the formation of NMJ synaptic boutons.

**Table 1:** Statistical tests used to analyze data

Feature	Comparison	Data structure (D’Agostino normality test)	Type of test	Sample size (# of NMJs, # of animals)	Mean	Statistic	*p* Value	Outlier test
Structure								
Bouton number	*w^1118^* vs *cow^GDP^/Df*	Normal	Unpaired two-tailed *t* test	w^1118^ (15,8); cow null (16,8)	23.53 vs 41.13	*t* = 8.296 df = 29	*p* < 0.0001	
Bouton number	*vglut-GAL4* vs *UAS-cow-RNAi*	Not normal	Kruskal–Wallis with Dunn's multiple-comparisons test	vglut-GAL4 (16,8); UAS-Cow-RNAi (16,8)	26.69 vs 28.8	Mean rank diff = −2.938	*p* > 0.9999	
	*vglut-GAL4* vs *vglut>cow-RNAi*			vglut-GAL4 (16,8); vglut>cow-RNAi (15,8)	26.69 vs 37.38	Mean rank diff = −19.09	*p* = 0.0002	
	*UAS-cow-RNAi* vs *vglut>cow-RNAi*			UAS-cow-RNAi (16,8); vglut>cow-RNAi (15,8)	28.8 vs 37.38	Mean rank diff = −16.16	*p* = 0.0031	
Bouton number	*24B-GAL4* vs *UAS-cow-RNAi*	Not normal	Kruskal–Wallis with Dunn's multiple-comparisons test	24B-GAL4 (16,8); UAS-cow-RNAi (16,8)	30.63 vs 31.5	Mean rank diff = −6.188	*p* = 0.6307	
	*24B-GAL4* vs *24B>cow-RNAi*			24B-GAL4 (16,8); 24B>cow-RNAi (16,8)	30.63 vs 28.06	Mean rank diff = 3.563	*p* > 0.9999	
	*UAS-cow-RNAi* vs *24B>cow-RNAi*			UAS-cow-RNAi (16,8); 24B>cow-RNAi (16,8)	31.5 vs 28.06	Mean rank diff = 9.75	*p* = 0.1451	
Bouton number	*vglut/+* vs *vglut>Cow*	Normal	Unpaired two-tailed *t* test	vglut/+ (16,8); vglut>Cow (16,8)	25.25 vs 27.06	*t* = 1.122 df = 30	*p* = 0.2706	
Bouton number	*24B/+* vs *24B>Cow*	Normal	Unpaired two-tailed *t* test	24B/+ (16,8); 24B>Cow (16,8)	30.38 vs 29.81	*t* = 0.2317 df = 30	*p* = 0.8183	
Bouton number	*FRT-Wg* vs *FRT-Wg;Cow^GDP^*	Not normal	Kruskal–Wallis with Dunn's multiple-comparisons test	FRT-Wg (24,12); FRT-Wg;Cow^GDP^ (24,12)	26.71 vs 31.71	Mean rank diff = −22.29	*p* = 0.0300	
	*FRT-Wg* vs *NRT-Wg*			FRT-Wg (24,12); NRT-Wg (24,12)	26.71 vs 27.04	Mean rank diff = −3.521	*p* > 0.9999	
	*FRT-Wg* vs *NRT-Wg;Cow^GDP^*			FRT-Wg (24,12); NRT-Wg;Cow^GDP^ (23,12)	26.71 vs 26.78	Mean rank diff = 0.4312	*p* > 0.9999	
	*FRT-Wg;Cow^GDP^* vs *NRT-Wg*			FRT-Wg;Cow^GDP^ (24,12); NRT-Wg (24,12)	31.71 vs 27.04	Mean rank diff = 18.77	*p* = 0.1085	
	*FRT-Wg;Cow^GDP^* vs *NRT-Wg;Cow^GDP^*			FRT-Wg;Cow^GDP^ (24,12); NRT-Wg;Cow^GDP^ (23,12)	31.71 vs 26.78	Mean rank diff = 22.72	*p* = 0.0278	
	*NRT-Wg* vs *NRT-Wg;Cow^GDP^*			NRT-Wg (24,12); NRT-Wg;Cow^GDP^ (23,12)	27.04 vs 26.78	Mean rank diff = 3.952	*p* > 0.9999	
Bouton number	*w^1118^* vs *cow^GDP^/+*	Not normal	Kruskal–Wallis with Dunn's multiple-comparisons test	w^1118^ (15,8); cow^GDP^/+ (15,8)	28.33 vs 35.73	Mean rank diff = −15.93	*p* = 0.0929	
	*w^1118^* vs *Notum^KO^/+*			w^1118^ (15,8); Notum^KO^/+ (16,8)	28.33 vs30.75	Mean rank diff = −5.565	*p* > 0.9999	
	*w^1118^* vs. *cow^GDP^/Notum^KO^*			w^1118^ (15,8); cow^GDP^/Notum^KO^ (16,8)	28.33 vs 46.13	Mean rank diff = −35.81	*p* < 0.0001	
	*cow^GDP^/+* vs *Notum^KO^/+*			cow^GDP^/+ (15,8); Notum^KO^/+ (16,8)	35.73 vs 30.75	Mean rank diff = 10.37	*p* = 0.6569	
	*cow^GDP^/+* vs *cow^GDP^/Notum^KO^*			cow^GDP^/+ (15,8); cow^GDP^/Notum^KO^ (16,8)	35.75 vs 46.13	Mean rank diff = −19.88	*p* = 0.0129	
	*Notum^KO^/+* vs *cow^GDP^/Notum^KO^*			Notum^KO^/+ (16,8); cow^GDP^/Notum^KO^ (16,8)	30.75 vs 46.13	Mean rank diff = −30.25	*p* < 0.0001	
Bouton number	*w^1118^* vs *cow^GDP^/cow^GDP^*	Normal	Ordinary one-way ANOVA with Tukey's multiple-comparisons test	w^1118^ (18,10); cow^GDP^/cow^GDP^ (19,10)	22.94 vs 33.74	*q* = 9.731 df = 76	*p* < 0.0001	
	*w^1118^* vs *Notum^KO^/Notum^KO^*			w^1118^ (18,10); Notum^KO^/Notum^KO^ (20,10)	22.94 vs 30.5	*q* = 6.897 df = 76	*p* < 0.0001	
	*w^1118^* vs *cow^GDP^,Notum^KO^/cow^GDP^,Notum^KO^*			w^1118^ (18,10); cow^GDP^,Notum^KO^/cow^GDP^,Notum^KO^ (23,12)	22.94 vs 29.13	*q* = 5.83 df = 76	*p* = 0.0005	
	*cow^GDP^/cow^GDP^* vs *Notum^KO^/Notum^KO^*			cow^GDP^/cow^GDP^ (19,10); Notum^KO^/Notum^KO^ (20,10)	33.74 vs 30.5	*q* = 2.996 df = 76	*p* = 0.1564	
	*cow^GDP^/cow^GDP^* vs *cow^GDP^,Notum^KO^/cow^GDP^,Notum^KO^*			cow^GDP^/cow^GDP^ (19,10); cow^GDP^,Notum^KO^/cow^GDP^,Notum^KO^ (23,12)	33.74 vs 29.13	*q* = 4.407 df = 76	*p* = 0.0135	
	*Notum^KO^/Notum^KO^* vs *cow^GDP^,Notum^KO^/cow^GDP^,Notum^KO^*			Notum^KO^/Notum^KO^ (20,10); cow^GDP^,Notum^KO^/cow^GDP^,Notum^KO^ (23,12)	30.5 vs 29.13	*q* = 1.328 df = 76	*p* = 0.7838	
% Satellite Boutons	*w^1118^* vs *cow^GDP^/Df*	Normal	Unpaired two-tailed *t* test	w^1118^ (15,8); cow null (15,8)	3.301 vs 3.336%	*t* = 0.03021 df = 28	*p* = 0.9761	ROUT, *Q* = 1%, removed 1 *cow^GDP^/Df* value
% Satellite boutons	*vglut-GAL4/+* vs *UAS-Cow-RNAi/+*	Normal	Ordinary one-way ANOVA with Tukey's multiple-comparisons test	vglut-GAL4/+ (16,8); UAS-Cow-RNAi/+ (15,8)	2.895 vs 2.908%	*q* = 0.016 df = 42	*p* > 0.9999	ROUT, iQ = 1%, removed 2 *vglut>Cow-RNAi* values
	*vglut-GAL4/+* vs *vglut>Cow-RNAi*			vglut-GAL4/+ (16,8); vglut>Cow-RNAi (14,8)	2.895 vs 5.772%	*q* = 3.309 df = 42	*p* = 0.0612	
	*UAS-Cow-RNAi/+* vs *vglut>Cow-RNAi*			UAS-Cow-RNAi/+ (15,8); vglut>Cow-RNAi (14,8)	2.908 vs 5.772%	*q* = 3.244 df = 42	*p* = 0.0677	
% Satellite boutons	*24B-GAL4/+* vs *UAS-Cow-RNAi/+*	Not normal	Kruskal–Wallis with Dunn's multiple-comparisons test	24B-GAL4/+ (16,8); UAS-Cow-RNAi/+ (16,8)	0.88 vs 2.381%	Mean rank diff = −8.656	*p* = 0.1328	
	*24B-GAL4/+* vs *24B>cow-RNAi*			24B-GAL4/+ (16,8); 24B>cow-RNAi (16,8)	0.88 vs 2.806%	Mean rank diff = −8.969	*p* = 0.1114	
	*UAS-Cow-RNAi/+* vs *24B>cow-RNAi*			UAS-Cow-RNAi/+ (16,8): 24B>cow-RNAi (16,8)	2.381 vs 2.806%	Mean rank diff = −0.3125	*p* > 0.9999	
% Satellite boutons	*vglut/+* vs *vglut>Cow*	Not normal	Mann–Whitney test	vglut/+ (16,8); vglut>Cow (16,8)	2.326 vs 7.121%	*U* = 38	*p* = 0.0003	
% Satellite boutons	*24B/+* vs *24B>Cow*	Normal	Unpaired two-tailed *t* test	24B/+ (16,8); 24B>Cow (16,8)	3.164 vs 5.476%	*t* = 1.177 df = 30	*p* = 0.2486	
% Satellite boutons	*FRT-Wg* vs *FRT-Wg;Cow^GDP^*	Not normal	Kruskal–Wallis with Dunn's multiple-comparisons test	FRT-Wg (16,8); FRT-Wg;Cow^GDP^ (16,8)	2.038 vs 1.002%	Mean rank diff = 5.167	*p* > 0.9999	
	*FRT-Wg* vs *NRT-Wg*			FRT-Wg (16,8); NRT-Wg (16,8)	2.038 vs 8.304%	Mean rank diff = −26.08	*p* = 0.0021	
	*FRT-Wg* vs *NRT-Wg;Cow^GDP^*			FRT-Wg (16,8); NRT-Wg;Cow^GDP^ (16,8)	2.038 vs 3.595%	Mean rank diff = −5.452	*p* > 0.9999	
	*FRT-Wg;Cow^GDP^* vs *NRT-Wg*			FRT-Wg;Cow^GDP^ (16,8); NRT-Wg (16,8)	1.002 vs 8.304%	Mean rank diff = −31.25	*p* = 0.0001	
	*FRT-Wg;Cow^GDP^* vs *NRT-Wg;Cow^GDP^*			FRT-Wg;Cow^GDP^ (16,8); NRT-Wg;Cow^GDP^ (16,8)	1.002 vs3.595%	Mean rank diff = −10.62	*p* > 0.9999	
	*NRT-Wg* vs *NRT-Wg;Cow^GDP^*			NRT-Wg (16,8); NRT-Wg;Cow^GDP^ (16,8)	8.304 vs 3.595%	Mean rank diff = 20.63	*p* = 0.0038	
% Satellite boutons	*w^1118^* vs *cow^GDP^/+*	Not normal	Kruskal–Wallis with Dunn's multiple-comparisons test	w^1118^ (15,8); cow^GDP^/+ (15,8)	1.89 vs 3.079%	Mean rank diff = −7.867	*p* > 0.9999	
	*w^1118^* vs *Notum^KO^/+*			w^1118^ (15,8); Notum^KO^/+ (16,8)	1.89 vs 3.379%	Mean rank diff = −10.95	*p* = 0.4586	
	*w^1118^* vs *cow^GDP^/Notum^KO^*			w^1118^ (15,8); cow^GDP^/Notum^KO^ (16,8)	1.89 vs 3.337%	Mean rank diff = −13.2	*p* = 0.1961	
	*cow^GDP^/+* vs *Notum^KO^/+*			cow^GDP^/+ (15,8); Notum^KO^/+ (16,8)	3.079 vs 3.379%	Mean rank diff = −3.079	*p* > 0.9999	
	*cow^GDP^/+* vs *cow^GDP^/Notum^KO^*			cow^GDP^/+ (15,8); cow^GDP^/Notum^KO^ (16,8)	3.079 vs 3.337%	Mean rank diff = −5.329	*p* > 0.9999	
	*Notum^KO^/+* vs *cow^GDP^/Notum^KO^*			Notum^KO^/+ (16,8); cow^GDP^/Notum^KO^ (16,8)	3.379 vs 3.337%	Mean rank diff = −2.25	*p* > 0.9999	
% Satellite boutons	*w^1118^* vs *cow^GDP^/cow^GDP^*	Not normal	Kruskal–Wallis with Dunn's multiple-comparisons test	w^1118^ (18,10); cow^GDP^/cow^GDP^ (19,10)	1.904 vs 1.623%	Mean rank diff = 2.006	*p* > 0.9999	
	*w^1118^* vs *Notum^KO^/Notum^KO^*			w^1118^ (18,10); Notum^KO^/Notum^KO^ (20,10)	1.904 vs 2.443%	Mean rank diff = −1.989	*p* > 0.9999	
	*w^1118^* vs *cow^GDP^,Notum^KO^/cow^GDP^,Notum^KO^*			w^1118^ (18,10); cow^GDP^,Notum^KO^/cow^GDP^,Notum^KO^ (23,12)	1.904 vs 0.5223%	Mean rank diff = 9.155	*p* = 0.7029	
	*cow^GDP^/cow^GDP^* vs *Notum^KO^/Notum^KO^*			cow^GDP^/cow^GDP^ (19,10); Notum^KO^/Notum^KO^ (20,10)	1.623 vs 2.443%	Mean rank diff = −3.995	*p* > 0.9999	
	*cow^GDP^/cow^GDP^* vs *cow^GDP^,Notum^KO^/cow^GDP^,Notum^KO^*			cow^GDP^/cow^GDP^ (19,10); cow^GDP^,Notum^KO^/cow^GDP^,Notum^KO^ (23,12)	1.623 vs 0.5223%	Mean rank diff = 7.149	*p* > 0.9999	
	*Notum^KO^/Notum^KO^* vs *cow^GDP^,Notum^KO^/cow^GDP^,Notum^KO^*			Notum^KO^/Notum^KO^ (20,10); cow^GDP^,Notum^KO^/cow^GDP^,Notum^KO^ (23,12)	2.443 vs 0.5223%	Mean rank diff = 11.14	*p* = 0.2978	
Expression								
Cow intensity	*vglut/+* vs *vglut>Cow*	Not normal	Mann–Whitney test	vglut/+ (16,8); vglut>Cow (16,8)	1 vs 3.035	*U* = 0	*p* < 0.0001	
Cow intensity	*24B/+* vs *24B>Cow*	Not normal	Mann–Whitney test	24B/+ (16,8); 24B>Cow (16,8)	1 vs 3.907	*U* = 0	*p* < 0.0001	
Wg intensity	*vglut/+* vs *vglut>Cow*	Not normal	Mann–Whitney test	vglut/+ (16,8); vglut>Cow (16,8)	1 vs 0.6731	*U* = 46	*p* = 0.0014	
Wg intensity	*24B/+* vs *24B>Cow*	Normal	Unpaired two-tailed *t* test	24B/+ (16,8); 24B>Cow (16,8)	1 vs 1.518	*t* = 3.266 df = 30	*p* = 0.0027	
Wg intensity	*w^1118^* vs *cow^GDP^/+*	Normal	Ordinary one-way ANOVA with Tukey's multiple-comparisons test	w^1118^ (15,8); cow^GDP^/+ (15,8)	1 vs 0.885	*q* = 1.328 df = 56	*p* = 0.7840	
	*w^1118^* vs *Notum^KO^/+*			w^1118^ (15,8); Notum^KO^/+ (15,8)	1 vs 1.095	*q* = 1.094 df = 56	*p* = 0.8660	
	*w^1118^* vs *cow^GDP^/Notum^KO^*			w^1118^ (15,8); cow^GDP^/Notum^KO^ (15,8)	1 vs 0.9014	*q* = 1.139 df = 56	*p* = 0.8515	
	*cow^GDP^/+* vs *Notum^KO^/+*			cow^GDP^/+ (15,8); Notum^KO^/+ (15,8)	0.885 vs 1.095	*q* = 2.422 df = 56	*p* = 0.3268	
	*cow^GDP^/+* vs *cow^GDP^/Notum^KO^*			cow^GDP^/+ (15,8); cow^GDP^/Notum^KO^ (15,8)	0.885 vs 0.9014	*q* = 0.1886 df = 56	*p* = 0.9991	
	*Notum^KO^/+* vs *cow^GDP^/Notum^KO^*			Notum^KO^/+ (15,8); cow^GDP^/Notum^KO^ (15,8)	1.095 vs 0.9014	*q* = 2.234 df = 56	*p* = 0.3985	
Brp punctae number	*w^1118^* vs *cow^GDP^*	Normal	Unpaired two-tailed *t* test	w^1118^ (15,8); cow^GDP^ (15,8)	193.1 vs 284.8	*t* = 6.152 df = 28	*p* < 0.0001	
Brp punctae Volume	*w^1118^* vs *cow^GDP^*	Normal	Unpaired two-tailed *t* test	w^1118^ (15,8); cow^GDP^ (15,8)	0.8576 vs 0.7164 μm^3^	*t* = 3.429 df = 28	*p* = 0.0019	ROUT, *Q* = 1%, removed 1 *cow^GDP^* value
Brp punctae number	*w^1118^* vs *cow^GDP^*	Normal	Unpaired two-tailed *t* test	w^1118^ (11,8); cow^GDP^ (10,8)	298.6 vs 387.9	*t* = 3.598 df = 19	*p* = 0.0019	
GluR cluster number	*w^1118^* vs *cow^GDP^*	Normal	Unpaired two-tailed *t* test	w^1118^ (11,8); cow^GDP^ (9,6)	382 vs 542.8	*t* = 4.353 df = 18	*p* = 0.0004	
Function								
EJC amplitude	*w^1118^* vs *cow^GDP^*	Normal	Ordinary one-way ANOVA with Tukey's multiple-comparisons test	w^1118^ (26,20); cow^GDP^ (20,18)	171.6 vs 212.1 nA	*q* = 3.868 df = 53	*p* = 0.0227	ROUT, *Q* = 1%, removed 1 *cow^GDP^* value
	*w^1118^* vs *cow^GDP^/Df*			w^1118^ (26,20); cow^GDP^/Df (10,9)	171.6 vs 254.2 nA	*q* = 4.197 df = 53	*p* = 0.0123	
	*cow^GDP^* vs *cow^GDP^/Df*			cow^GDP^ (20,18); cow^GDP^/Df (10,9)	212.1 vs 254.2 nA	*q* = 1.063 df = 53	*p* = 0.7341	
EJC amplitude	*w^1118^* vs *cow^GDP^/+*	Normal	Ordinary one-way ANOVA with Tukey's multiple-comparisons test	w^1118^ (10,6); cow^GDP^/+ (11,6)	217.2 vs 234.9 nA	*q* = 0.9383 df = 40	*p* = 0.9101	
	*w^1118^* vs *notum^KO^/+*			w^1118^ (10,6); notum^KO^/+ (11,9)	217.2 vs 214.1 nA	*q* = 0.1649 df = 40	*p* = 0.9994	
	*w^1118^* vs *cow^GDP^/notum^KO^*			w^1118^ (10,6); cow^GDP^/notum^KO^ (12,7)	217.2 vs 235.9 nA	*q* = 1.009 df = 40	*p* = 0.8911	
	*cow^GDP^/+* vs *notum^KO^/+*			cow^GDP^/+ (11,6); notum^KO^/+ (11,9)	234.9 vs 214.1 nA	*q* = 1.13 df = 40	*p* = 0.8543	
	*cow^GDP^/+* vs *cow^GDP^/notum^KO^*			cow^GDP^/+ (11,6); cow^GDP^/notum^KO^ (12,7)	234.9 vs 235.9 nA	*q* = 0.05304 df = 40	*p* > 0.9999	
	*notum^KO^/+* vs *cow^GDP^/notum^KO^*			notum^KO^/+ (11,9); cow^GDP^/notum^KO^ (12,7)	214.1 vs 235.9 nA	*q* = 1.208 df = 40	*p* = 0.8282	
mEJC Frequency	*w^1118^* vs *cow^GDP^*	Normal	Ordinary one-way ANOVA with Tukey's multiple-comparisons test	w^1118^ (22,17); cow^GDP^ (21,15)	1.396 vs 1.765 Hz	*q* = 1.419 df = 53	*p* = 0.5780	
	*w^1118^* vs *cow^GDP^/Df*			w^1118^ (22,17); cow^GDP^/Df (13,11)	1.396 vs 2.41 Hz	*q* = 3.406 *q* = 53	*p* = 0.0503	
	*cow^GDP^* vs *cow^GDP^/Df*			cow^GDP^ (21,15); cow^GDP^/Df (13,11)	1.764 vs 2.41 Hz	*q* = 2.15 df = 53	*p* = 0.2897	
mEJC Frequency	*vglut-GAL4/+* vs *vglut>Cow-RNAi*	Normal	Unpaired two-tailed *t* test	vglut-GAL4/+ (10,7); vglut>Cow-RNAi (11,7)	1.497 vs 2.449 Hz	*t* = 2.142 df = 19	*p* = 0.0454	ROUT, *Q* = 1%, removed 1 vglut-GAL4/+ value
mEJC amplitude	*w^1118^* vs *cow^GDP^*	Normal	Ordinary one-way ANOVA with Tukey's multiple-comparisons test	w^1118^ (21,16); cow^GDP^ (21,15)	0.7518 vs 0.8682 nA	*q* = 2.506 df = 52	*p* = 0.1889	ROUT, *Q* = 1%, removed 1 *w^1118^* value
	*w^1118^* vs *cow^GDP^/Df*			w^1118^ (21,16); cow^GDP^/Df (13,11)	0.7518 vs 0.7165 nA	*q* = 0.6647 df = 52	*p* = 0.8856	
	*cow^GDP^* vs *cow^GDP^/Df*			cow^GDP^ (21,15); cow^GDP^/Df (13,11)	0.8682 vs 0.7165 nA	*q* = 2.857 df = 52	*p* = 0.1175	
mEJC amplitude	*vglut-GAL4/+* vs *vglut>Cow-RNAi*	Normal	Unpaired two-tailed *t* test	vglut-GAL4/+ (11,7); vglut>Cow-RNAi (11,7)	0.8015 vs 0.8446 nA	*t* = 0.8011 df = 20	*p* = 0.4325	
Frequency	*vglut/+* vs *vglut>RNAi*	Not normal (Shapiro–Wilk normality test performed because N too small)	Mann–Whitney test	vglut/+ (7,4); vglut>RNAi (6,3)	1.617 vs 2.977 Hz/μm^2^	*U* = 7	*p* = 0.0513	
Mean Δ*F*/*F*_0_	*vglut/+* vs *vglut>RNAi*	Normal (Shapiro–Wilk normality test performed because N too small)	Unpaired two-tailed *t* test	vglut/+ (8,4); vglut>RNAi (5,3)	0.7912 vs 1.058 ΔF/F_0_	*t* = 3.013 df = 11	*p* = 0.0118	

**Figure 4.**
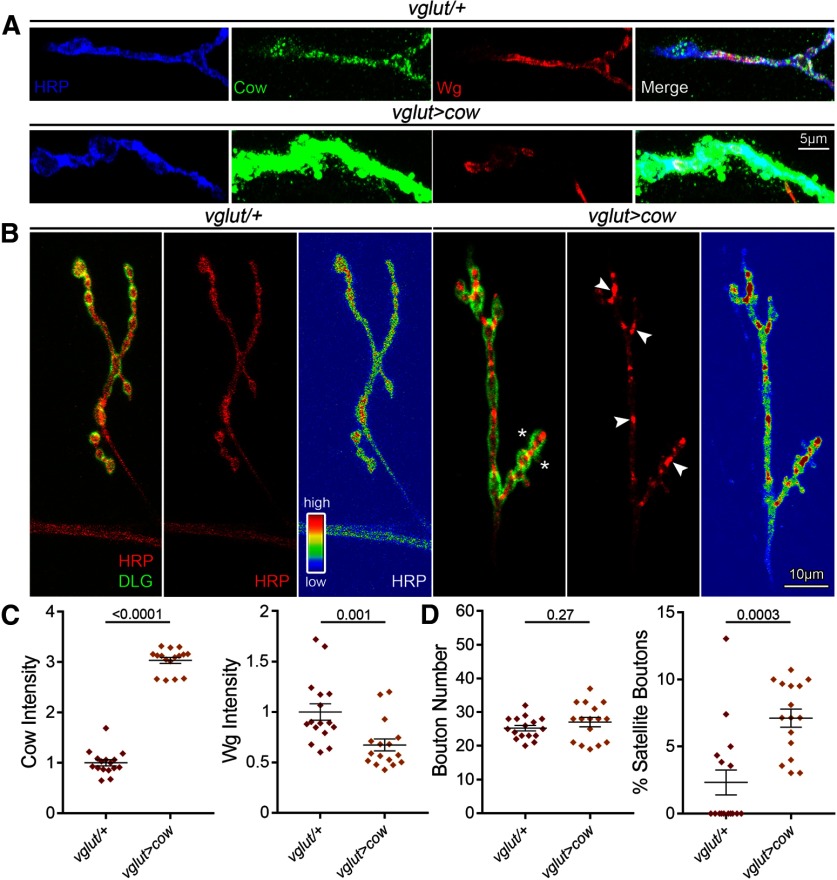
Presynaptic Cow elevation decreases Wg and increases satellite boutons. ***A***, Confocal images of NMJ boutons triple labeled with anti-HRP (blue), anti-Cow (green), and anti-Wg (red), and merged (far right) comparing transgenic controls (*vglut-Gal4/+*) to motor neuron Cow overexpression condition (*vglut>cow*). Labeling was done in the absence of detergent to reveal only secreted Cow and Wg. ***B***, Representative muscle 4 NMJ images colabeled for presynaptic HRP (red) and postsynaptic DLG (green) comparing controls (*vglut-Gal4/+*) to neuronal Cow overexpression (*vglut>cow*). Asterisks indicate satellite boutons. The second panel shows HRP alone with arrows indicating distinct spots of HRP accumulation, and the third panel shows HRP expression heatmap. ***C***, Quantification of confocal fluorescence intensity for Cow (left) and Wg (right) in the two conditions shown in a scatter plot, with mean ± SEM. ***D***, Quantification of synaptic bouton number (left) and the percentage of satellite boutons (right) in transgenic controls versus *cow* neuronal overexpression shown in a scatter plot, with mean ± SEM. *p* Values are shown for each statistical comparison.

When Cow is overexpressed in motor neurons (*vglut-*Gal4>*UAS-Cow*), Cow is elevated at the NMJ with a concomitant decrease in extracellular Wg ligand (Fig. 4*A*). The NMJs have a typical number of mature boutons, but an increase in satellite boutons (Fig. 4*B*). Interestingly, *cow* neuronal OE causes HRP redistribution with distinct spots of accumulation (Fig. 4*B*, heatmap on right). Quantification shows a significant increase in Cow levels secreted at the NMJ terminal (normalized *vglut*-Gal4*/+*, 1.0 ± 0.06 vs *vglut>cow*, 3.04 ± 0.06; *p* < 0.0001), with a significant decrease in extracellular Wg levels (*vglut*-Gal4*/+*, 1.0 ± 0.08 vs *vglut>cow*, 0.67 + 0.06; *p* = 0.001; Fig. 4*C*). Quantification shows no change in bouton number (*vglut*-Gal4*/+*, 25.25 ± 0.81 vs *vglut>Cow*, 27.06 ± 1.4; *p* = 0.27), but a significant increase in satellite boutons (*vglut*-Gal4*/+*, 2.33 ± 0.94% vs *vglut>cow*, 7.12 ± 0.67; *p* = 0.0003; Fig. 4*D*). Whereas neuronal *cow* OE elevates normal Cow expression at the NMJ, muscle *cow* OE causes aberrant, ectopic expression (normalized *24B*-Gal4*/+*, 1.0 ± 0.03 vs *24B>cow*, 3.91 ± 0.23; *p* < 0.0001), which increases Wg ligand (*24B*-Gal4*/+*, 1.0 ± 0.07 vs *24B>Cow*, 1.52 ± 0.14*; p* = 0.003). Muscle-targeted *cow* OE causes no change in mature boutons (*24B*-Gal4*/+*, 30.38 ± 1.94 vs *24B>cow*, 29.81 ± 1.46; *p* = 0.82) or the percentage of satellite boutons (*24B*-Gal4*/+*, 3.16 ± 1.16% vs *24B>cow*, 5.48 ± 1.58; *p* = 0.2486). We next assayed synaptic functional differentiation to test whether these structural changes have functional consequences.

### Cow restricts presynaptic vesicle fusion and neurotransmission strength

**Figure 5.**
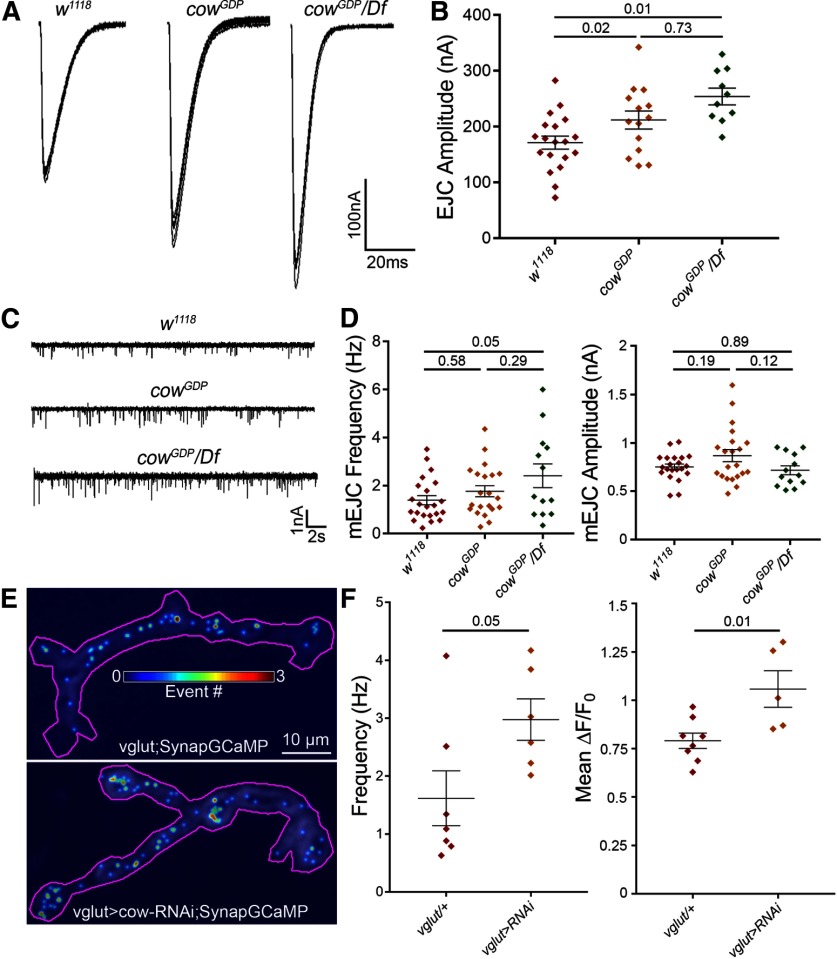
Presynaptic Cow limits synaptic vesicle fusion for NMJ neurotransmission. ***A***, Representative motor nerve stimulation-evoked EJC traces (1.5 mm [Ca^2+^]) from the *w^1118^* genetic background control, *cow^GDP^* homozygous mutant, and *cow^GDP^*/*Df* mutant. ***B***, Quantification of EJC amplitudes in the three genotypes shown in a scatter plot, with mean ± SEM. ***C***, Representative mEJC recording traces from the same genotypes. ***D***, Quantification of mEJC frequency (left) and amplitude (right) from the three genotypes. ***E***, Representative probability maps (30 s) of SynapGCaMP imaging of mEJC events in motor neuron-targeted Gal4 driver control (*vglut*-Gal4/+, top) and *cow* RNAi knockdown (*vglut*>*cow*-RNAi, bottom), indicating mEJC location (dot) and frequency (color; see scale inset). ***F***, Quantification of SynapGCaMP event frequency (in Hz/μm^2^; left) and fluorescence intensity (Δ*F*/*F*_0_; right) shown in scatter plots, with mean ± SEM. *p* Values are shown for each statistical comparison.

We used the following two methods to assay NMJ synaptic functional differentiation and neurotransmission strength: (1) TEVC electrophysiology (Dani et al., 2012; Parkinson et al., 2013; Kopke et al., 2017); and (2) imaging genetically encoded calcium reporter SynapGCaMP6f (Newman et al., 2017). For assaying evoked transmission, muscle 6 was clamped (−60 mV), while the motor nerve was stimulated with a suction electrode (1.5 mm [Ca^2+^]). EJC traces were recorded (0.2 Hz, 10 consecutive stimuli) to measure the average amplitude. For assaying mEJC events, spontaneous synaptic vesicle fusions were recorded, measuring frequency and amplitude. The mEJC frequency indicates presynaptic vesicular release (number of active synapses, fusion probability), and mEJC amplitude indicates number of activated postsynaptic receptors. For quantal imaging, the SynapGCaMP reporter (*MHC-CD8-GCaMP6f-Sh*) contains a myosin heavy chain (MHC) promoter for muscle targeting, CD8 transmembrane domain for membrane targeting, and Shaker (Sh) K^+^ channel C-terminal tail for postsynaptic targeting (Newman et al., 2017). By imaging transmission, we are able to specifically determine the changes in quantal activity at the convergent motor neuron inputs separately. Live-imaging recordings were made of the SynapGCaMP reporter at muscle 4, with spontaneous event frequency divided by the NMJ synaptic area, and event amplitude measured as the change in the fluorescence signal over the baseline NMJ fluorescence (Δ*F*/*F*_0_). Representative recordings and summarized data are shown in Figure 5.

With nerve stimulation, evoked transmission is clearly and consistently increased in *cow* nulls compared with *w^1118^*controls (Fig. 5*A*). Quantified measurements show EJC amplitude significantly elevated (*w^1118^*, 175.4 ± 9.93 nA vs *cow^GDP^*, 214.6 ± 12.24; *p* = 0.023; *w^1118^*, 175.4 ± 9.93 vs *cow^GDP^/Df*, 254.2 ± 14.99; *p* = 0.012; Fig. 5*B*). Although the *cow^GDP^/Df* mutants show a slight increase in mEJC frequency, no change was observed in the *cow^GDP^* nulls. We found no change in amplitude (Fig. 5*C*). In quantified measurements, mEJC frequency is slightly increased in homozygous mutants and more increased in the *cow^GDP^/Df* (*w^1118^*, 1.396 ± 0.19 Hz vs *cow^GDP^/cow^GDP^*, 1.764 ± 0.23; *p* = 0.58; *w^1118^*, 1.396 ± 0.19 vs *cow^GDP^/Df*, 2.41 ± 0.49; *p* = 0.05; Fig. 5*D*, left). There is no significant change in mEJC amplitude (*w^1118^*, 0.75 ± 0.03 nA vs *cow^GDP^/cow^GDP^*, 0.87 ± 0.06; *p* = 0.189; *w^1118^*, 0.75 ± 0.03 nA vs *cow^GDP^/Df*, 0.72 ± 0.05; *p* = 0.886; Fig. 5*D*, right). Neuronally targeted *cow*-RNAi causes an increase in mEJC frequency (*vglut*-Gal4*/+*, 1.5 ± 0.33 Hz vs *vglut>Cow-RNAi*, 2.45 ± 0.3; *p* = 0.045), but not amplitude (*vglut*-Gal4*/+*, 0.8 ± 0.03 nA vs *vglut> Cow-RNAi*, 0.85 ± 0.42; *p* = 0.4325). SynapGCaMP imaging also shows increased fusion frequency in type Ib boutons (Fig. 5*E*). In quantal imaging measurements, spontaneous fusion frequency increases (*vglut*-Gal4*/+*, 1.62 ± 0.47 Hz/μm^2^ vs *vglut>cow-RNAi*, 2.98 ± 0.36; *p* = 0.051; Fig. 5*F*, left). Interestingly, event magnitude also significantly increases (*vglut*-Gal4*/+*, 0.79 ± 0.04 Δ*F*/*F*_0_ vs *vglut>cow-RNAi*, 1.06 ± 0.09; *p* = 0.012; Fig. 5*F*, right). These results demonstrate that Cow limits evoked neurotransmission strength and suggest that neuronally secreted Cow regulates synaptic vesicle fusion at the presynaptic active zone.

### Cow restricts presynaptic active zone and glutamatergic synapse formation

**Figure 6.**
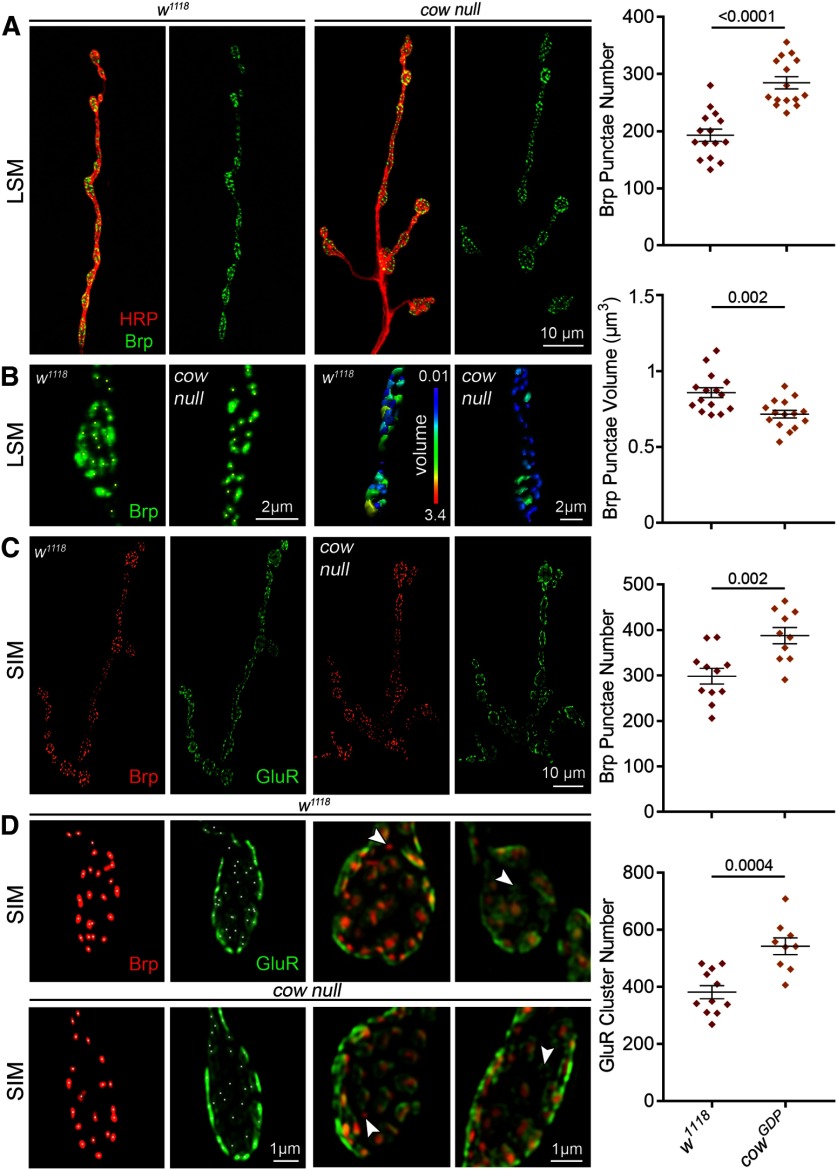
Cow limits presynaptic active zones and glutamatergic synapse number. ***A***, Representative muscle 4 NMJ images from confocal LSM of genetic background controls (*w^1118^*, left) and cow null mutants (*cow^GDP^*, right) colabeled for presynaptic membrane marker (HRP, red) and the active zone scaffold Brp (green). Brp alone is shown in right panels and the quantified Brp punctae number is shown to the right. ***B***, High-magnification synaptic bouton images with Brp punctate identified using Imaris software (asterisks, left) and volume indicated in a heatmap (scale, 0.01–3.4 μm^3^; right). Quantified Brp punctae volume shown to the right. ***C***, Representative NMJ images from a SIM of controls (*w^1118^*) and *cow* nulls (*cow^GDP^*) colabeled for both presynaptic active zones (Brp, red) and postsynaptic glutamate receptors (GluRIIC, green). The quantified Brp punctae number is shown to the right. ***D***, High-magnification SIM images of juxtaposed Brp punctae and GluR clusters at synapses. Arrowheads indicate Brp or GluR domains without a partner, which are observed at equal frequency in both genotypes. Quantified GluR cluster number is shown to the right. Data shown in scatter plots, with mean ± SEM. *p* Values are shown for each statistical comparison.

We next used imaging to assay presynaptic and postsynaptic molecular components of the synapse to test the hypothesis of increased NMJ synapse number in *cow* mutants. The presynaptic AZ is the specialized site of SV fusion that mediates the release of the glutamate neurotransmitter. Brp tethers both the voltage-gated Ca^2+^ channels and SVs to the AZ, and is the best AZ marker (Hallermann et al., 2010). Each AZ directly apposes a postsynaptic GluR cluster to mediate fast neurotransmission (Schuster et al., 1991). We used colabeling with both anti-Brp (Wagh et al., 2006) and anti-GluRIIC (aka GluRIII; Marrus et al., 2004) to compare *cow* null mutants to *w^1118^*genetic background controls (Fig. 6). Brp AZ punctae occur much more often in *cow* null NMJs (Fig. 6*A*), but are consistently smaller in volume (Fig. 6*B*). In quantified measurements, the number of Brp AZ punctae per NMJ is significantly increased in the *cow* null mutants compared with matched controls (*w^1118^*, 193.1 ± 10.55 vs *cow^GDP^*, 284.8 ± 10.54; *p* < 0.0001; Fig. 6*A*, right), but the average volume of the Brp AZ synaptic punctae is significantly decreased in the mutants (*w^1118^*, 0.86 ± 0.033 μm^3^ vs *cow^GDP^*, 0.72 ± 0.025; *p* = 0.0019; Fig. 6*B*, right). This is consistent with a previous report also showing a reciprocal relationship between Brp AZ punctae number and volume (Graf et al., 2009).

Brp AZ punctae are precisely juxtaposed to GluR clusters in a functional synapse (Menon et al., 2013). For better resolution to image postsynaptic GluR clusters and quantify the synaptic apposition, SIM was used (Gustafsson, 2000). To compare with previous LSM, Brp AZs were first measured to find a consistent increase in the *cow* null mutants, but with larger punctae numbers, presumably due to increased resolution (*w^111^*, 298.6 ± 17.2 vs *cow^GDP^*, 387.9 ± 17.86; *p* = 0.0019; Fig. 6*C*). There is also a similar increase in GluR clusters (*w^1118^*, 382 ± 23.21 vs *cow^GDP^*, 542.8 ± 29.41; *p* = 0.0004; Fig. 6*D*). Brp punctae and GluR clusters almost always partner, with rare exceptions seen at a similar frequency in controls and mutants (Fig. 6*D*). There are more GluR clusters than Brp punctae in both genotypes. The GluR/Brp ratio was measured to test for defects in synaptic apposition. If there is a larger ratio in the mutants compared with controls, this would indicate more GluR clusters without a Brp AZ. Conversely, a smaller ratio would indicate more GluR clusters paired with a presynaptic partner. Quantified measurements show no difference in the GluR/Brp ratio between controls and the *cow* null mutants (*w^1118^*, 1.29 ± 0.04 vs *cow^GDP^*, 1.36 ± 0.05; *p* = 0.272). Together, these results demonstrate that Cow limits NMJ synapse formation, which is consistent with strengthened neurotransmission.

### Membrane-tethering Wg prevents *cow* null defects in NMJ development

**Figure 7.**
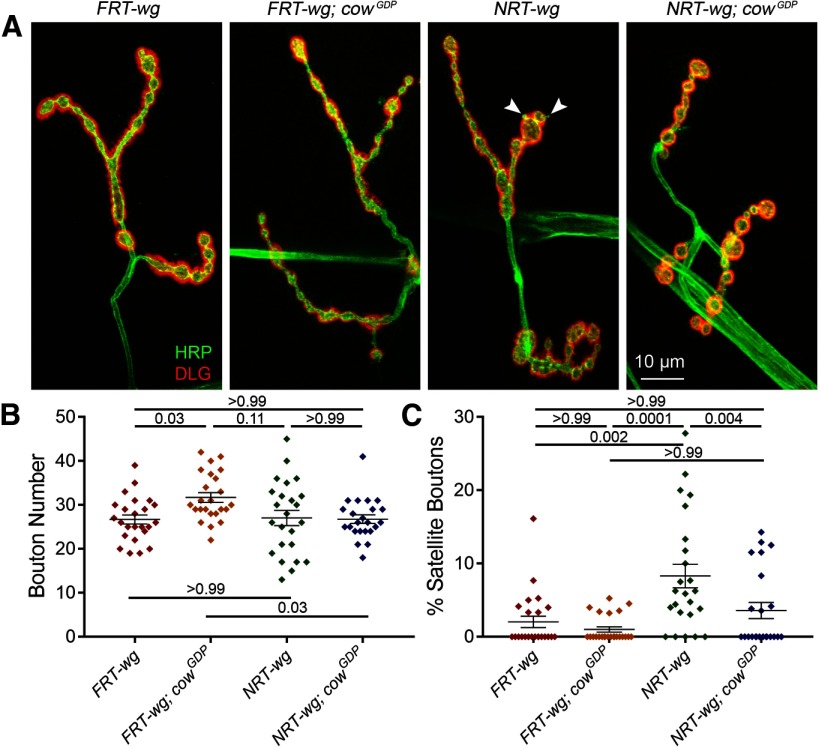
Membrane-tethered Wg prevents *cow* null defects in bouton formation. ***A***, Representative confocal images of muscle 4 NMJs colabeled with presynaptic HRP marker (green) and postsynaptic DLG marker (red) in Wg control (*FRT-wg*), cow null (*FRT-wg*; *cow^GDP^*), tethered Wg (*NRT-wg*), and tethered Wg in *cow* null background (*NRT-wg; cow^GDP^*). ***B***, ***C***, Quantification of total NMJ synaptic bouton number (*B*) and the percentage of satellite boutons (*C*) in the four genotypes shown in a scatter plot, with mean ± SEM. *p* Values are shown for each statistical comparison.

Our starting hypothesis was that Cow regulates Wg by binding the ligand in the extracellular space and carrying it across the synaptic cleft (from neuron to muscle). This hypothesis is based on published work demonstrating that Cow is secreted, directly binds secreted Wg and acts to mediate intercellular transport (Chang and Sun, 2014). To test this hypothesis, we obtained transgenic lines with the *wg* gene cut from its native locus via FRT sites and then replaced either without (*FRT-wg*; transgenic control) or with (*NRT-wg*) a membrane tether. Importantly, HA-tagged *NRT-wg* is not secreted from Wg-expressing cells and fails to maintain the expression of long-range Wg targets (Alexandre et al., 2014). We tested whether tethering Wg to the membrane affects NMJ development. Comparing *FRT-wg* to *NRT-wg*, there is increased expression of the Wg ligand around presynaptic boutons (data not shown). To determine whether tethered Wg can bind Fz2 receptors, the NMJ bouton number was measured to assess presynaptic Wg signaling. Next, *NRT-wg* was combined with the *cow* null mutant (*NRT-wg; cow^GDP^*) to test the hypothesis that Cow normally acts to regulate secreted Wg function. If Wg needs to be secreted and transported dependent on Cow function, then *NRT-wg* and *NRT-wg; cow^GDP^* would be predicted to have the same phenotype. Representative images and summarized data are shown in Figure 7.

In comparing the control *FRT-wg* and tethered *NRT-wg*, there is no change in mature NMJ bouton number, but there is a clear increase in the number of immature satellite boutons when Wg is tethered (Fig. 7*A*). In quantified measurements, *NRT-wg* has the same number of NMJ synaptic boutons as the control (*FRT-wg*, 26.71 ± 1.04 vs *NRT-wg*, 27.04 ± 1.72; *p* = 0.999; Fig. 7*A*,*B*), but a fourfold increase in the percentage of satellite boutons (*FRT-wg*, 2.04 ± 0.77% vs *NRT-wg*, 8.3 ± 1.62; *p* = 0.0019; Fig. 7*C*). When membrane-tethered Wg is placed in the *cow* null background (*NRT-wg; cow^GDP^*), both the mature synaptic bouton number and the percentage of satellite boutons are similar to the *FRT-wg* control levels (Fig. 7*A*). In quantified measurements, the mature bouton number is no longer different between the two genotypes (*FRT-wg*, 26.71 ± 1.04 vs *NRT-wg; cow^GDP^*, 26.78 ± 0.97; *p* = 0.999; Fig. 7*B*; Table 1, all other comparisons), and the satellite boutons are also restored to near-normal levels (*FRT-wg*, 2.04 ± 0.77% vs *NRT-wg; cow^GDP^*, 3.60 ± 1.1; *p* = 0.999; Fig. 7*C*). Together, these results suggest that Cow facilitates Wg-dependent satellite bouton formation, and that Wg has to be secreted for Cow to act on it. However, in contrast to the original hypothesis, Cow acts as a negative regulator of secreted Wg signaling at the NMJ, suggesting that it should interact with other Wg-negative regulators in the extracellular synaptomatrix.

### Cow and Notum function together to restrict NMJ growth and bouton formation

**Figure 8.**
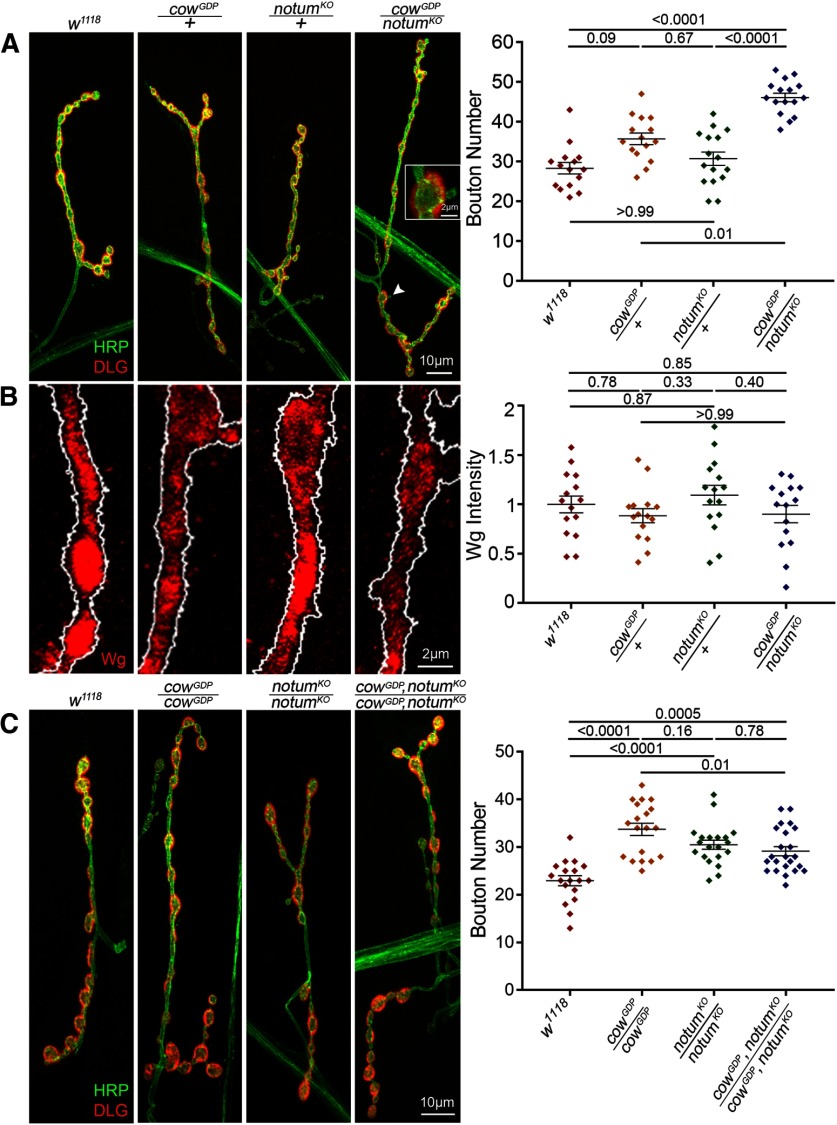
Cow and Notum act in the same Wg pathway to limit NMJ bouton number. ***A***, Confocal images of the muscle 4 NMJ colabeled with presynaptic HRP marker (green) and postsynaptic DLG marker (red) in the genetic background control (*w^1118^*), *cow* null heterozygote (*cow^GDP^/+*), *notum* null heterozygote (*notum^KO^/+*), and *cow/notum* transheterozygote (*cow^GDP^/notum^KO^*). Quantified bouton number is shown to the right. ***B***, High-magnification NMJ confocal images of anti-Wg labeling at synaptic boutons of the same indicated genotypes. The presynaptic HRP marker boundary is outlined in white. Quantified Wg fluorescence intensity is shown to the right, normalized to the background control (*w^1118^*). ***C***, Confocal images of the muscle 4 NMJ colabeled with presynaptic HRP marker (green) and postsynaptic DLG marker (red) in the genetic background control (*w^1118^*), *cow* null (*cow^GDP^/cow^GDP^*), *notum* null (*notum^KO^/notum^KO^*), and *cow/notum* double null (*cow^GDP^,notum^KO^/cow^GDP^,notum^KO^*). Quantified bouton number is shown to the right. Data shown in scatter plots, with mean ± SEM. *p* Values are shown for each statistical comparison.

The secreted deacylase Notum has also been recently shown to regulate NMJ synaptic bouton formation via the negative regulation of Wg trans-synaptic signaling (Kopke et al., 2017). Notum restricts Wnt signaling by cleaving the Wg palmitoyl group that binds to Fz2 receptors (Kakugawa et al., 2015). In *notum* null mutants, NMJ Wg signaling is elevated both presynaptically and postsynaptically, resulting in increased synaptic bouton number, synapse number, and neurotransmission strength (Kopke et al., 2017). To test the hypothesis that the increased NMJ development in *cow* null mutants is similarly caused by an increase in Wg trans-synaptic signaling, we performed the genetic test of combining *cow* and *notum* null heterozygotes to assay effects on NMJ synaptic bouton development. The failure of mutant alleles at two different loci to complement one another is one method to test for an *in vivo* interaction of the gene products in a common signaling mechanism (nonallelic noncomplementation; Yook et al., 2001; Hawley and Gilliland, 2006). In this case, the interaction tests the hypothesis that Cow and Notum have closely associated functions in the regulation of Wg synaptic signaling via direct interaction with the Wg ligand in the extracellular synaptomatrix. We compared bouton formation in genetic background control (*w^1118^*); *cow* null (*cow^GDP^*), and *notum* null (*notum^KO^*) homozygotes and heterozygotes; *cow/notum* trans*-*heterozygotes; and *cow/notum* double null mutant (*cow^GDP^,notum^KO^/cow^GDP^,notum^KO^*). Representative images and summarized data are shown in Figure 8.

The trans-heterozygote has a clearly expanded NMJ with more synaptic boutons compared with controls, as well as other *wg* mutant phenotypes such as the appearance of ghost boutons (Fig. 8*A*, inset). Ghost boutons are immature boutons that contain the HRP marker, but do not yet contain the postsynaptic DLG protein (Ataman et al., 2006). The *cow* (*cow^GDP^/+*) and *notum* (*notum^KO^*/+) heterozygotes alone are no different from *w^1118^*controls and lack synaptic features of impaired Wg signaling (Fig. 8*A*, Table 1). In quantified measurements, trans-heterozygotes have strongly increased bouton numbers (*w^1118^*, 28.33 ± 1.46 vs *cow^GDP^/notum^KO^*, 46.13 ± 1.08; *p* < 0.0001; Fig. 8*A*, right; Table 1, all other comparisons). Extracellular Wg labeling without cellular permeabilization in all these genotypes indicates no difference in the Wg fluorescence intensity (Fig. 8*B*). In quantified measurements, there is no detectable change in Wg ligand levels between controls and *cow/notum* trans-heterozygotes (normalized *w^1118^*, 1.0 ± 0.09 vs *cow^GDP^/*+; *notum^KO^/*+, 0.9 ± 0.09; *p* = 0.852; Fig. 8*B*, right; Table 1, all other comparisons). The double null mutants have significantly increased bouton numbers compared with controls but no increase compared with each null alone (*w^1118^*, 22.94 ± 1.05 vs *cow^GDP^*, *notum^KO^/cow^GDP^*,*notum^KO^*, 29.13 ± 0.97; *p* = 0.0005; Fig. 8*C*, right; Table 1, all other comparisons). Interestingly, trans-heterozygotes show no change in nerve stimulation-evoked EJC recordings (Table 1). These results indicate that Cow and Notum act in the same pathway to restrict Wg signaling in structural development, and that the level of extracellular Wg ligand alone is not predictive of signaling activity.

## Discussion

The function of signaling ligands in the extracellular space is tightly regulated to ensure coordinated intercellular development, often via glycan-dependent mechanisms (Dani and Broadie, 2012; Parkinson et al., 2013; Shilts and Broadie, 2017). The most recently discovered *Drosophila* HSPG, secreted Cow, was characterized with this role (Chang and Sun, 2014). In the developing wing disk, the Wnt Wg is produced in a stripe of cells at the dorsal/ventral margin boundary, and acts as an intercellular morphogen through Fz2 receptor signaling (Bhanot et al., 1996; Zecca et al., 1996; Neumann and Cohen, 1997). The glypican HSPGs Dally and Dlp, bound to outer plasma membrane leaflets via GPI anchors, bind Wg to regulate both ligand distribution and intercellular signaling (Tsuda et al., 1999; Baeg et al., 2001; Dani et al., 2012; Dear et al., 2017). It has been proposed that Dally/Dlp HSPGs are involved in the movement of extracellular Wg to form a morphogen gradient (Han et al., 2005). However, in *dally dlp* double mutant clones, extracellular Wg is detected far away from Wg-secreting cells, suggesting that another extracellular factor can transport Wg. Cow was shown to fill this role by binding extracellular Wg to increase stability and rate of movement from producing to receiving cells (Chang and Sun, 2014). Supporting this model, *cow* mutants manifest Wg ligand gain-of-function/overexpression phenotypes for short-range targets, and loss-of-function phenotypes for long-range targets.

At the NMJ, such a long-range Wg morphogen transport function is not seemingly required, except perhaps as a clearance mechanism, but Wg extracellular regulation and short-range Wg transport to cross the synaptic cleft is critical for NMJ development (Packard et al., 2002; Friedman et al., 2013; Dear et al., 2016; Parkinson et al., 2016). At the forming of NMJ, Wg from neurons and glia signals both presynaptically (neuronal) and postsynaptically (muscle) via Fz2 receptors (Packard et al., 2002; Kerr et al., 2014). In the motor neuron, Wg signaling inhibits the GSK3β homolog Sgg to regulate the MAP1B homolog Futsch to modulate microtubule dynamics controlling NMJ bouton formation (Miech et al., 2008). However, Futsch distribution and microtubule dynamics do not change with elevated Wg signaling (Kopke et al., 2017), so this pathway alone does not explain the increased bouton formation with increased Wg signaling. In the postsynaptic muscle, Wg signaling drives Fz2 endocytosis and C-terminus cleavage, with transport to the nucleus regulating mRNAs involved in synaptogenesis, including postsynaptic GluR distribution (Speese et al., 2012). In *wg* mutants, GluRs are more diffuse; with clusters irregular in size/shape, increased receptor numbers and a larger postsynaptic volume (Packard et al., 2002; Speese et al., 2012; Kerr et al., 2014). Thus, Wg trans-synaptic signaling controls both NMJ structure and function.

Based on the findings from Chang and Sun (2014), we hypothesized that Cow binds Wg to facilitate the transport across the synapse to Fz2 receptors on the muscle. If this is correct, we would expect a presynaptic Wg OE phenotype in the absence of Cow (Wg buildup at the source), and a postsynaptic Wg decrease/loss phenotype (failure of Wg transport). Presynaptically, we find increased synaptic bouton number in *cow* null mutants phenocopying the Wg OE condition (Kopke et al., 2017), consistent with this hypothesis. These results indicate that Cow normally inhibits NMJ bouton formation, consistent with the effects of inhibiting presynaptic Wg signaling (Packard et al., 2002). Postsynaptically, we find an increased number of GluR clusters due to elevated synapse formation in *cow* null mutants, but no evidence of diffuse GluR clusters of irregular size/shape and larger volume, as has been reported in *wg* mutants (Packard et al., 2002; Speese et al., 2012; Kerr et al., 2014). Therefore, we do not find strong support for the second prediction of the hypothesis. GluR changes within single postsynaptic domains are challenging to see even with enhanced resolution microscopy (e.g., the SIM used here; Gustafsson, 2000), but future studies could focus more on GluRIIA cluster size/shape/intensity in *cow* mutants. If GluR defects are detected in *cow* nulls, it would be interesting to test the FNI pathway (Mathew et al., 2005).

Wg signaling regulates multiple steps of NMJ development including branching, satellite bouton budding, and synaptic bouton maturation (Koles and Budnik, 2012). None of the *cow* manipulations cause changes in branching, indicating that Cow does not regulate this Wg signaling, likely working in concert with other Wg regulators. Wg loss (*wg^ts^*) decreases bouton formation (Packard et al., 2002), while neural Wg OE increases branching, satellite, and total bouton numbers (Packard et al., 2002; Miech et al., 2008; Kopke et al., 2017). Satellite boutons represent an immature stage of development, with small boutons connected to the mature (parent) bouton or adjacent axon (Torroja et al., 1999; Gatto and Broadie, 2008). Neuronal Cow OE does not change mature bouton number, but increases satellite bouton budding. Neuronal Cow RNAi also increases satellite boutons. Thus, changing neural Cow levels in either direction elevates satellite bouton numbers, suggesting different consequences on budding versus developmental arrest. It also appears that the cellular source of secreted Cow, or the balance between sources, may be important for proper Wg regulation. Importantly, glia-secreted Wg regulates distinct aspects of synaptic development (Kerr et al., 2014), with loss of glial-derived Wg accounting for some, but not all, of *wg* mutant phenotypes. Similarly, cell-targeted *cow* manipulations cause different NMJ phenotypes. There is no evidence for normal Cow function in postsynaptic muscle, but it remains possible that Cow secreted from glia could regulate Wg trans-synaptic signaling.

Increasing Wg signaling elevates evoked transmission strength and functional synapse number (Kopke et al., 2017), which is phenocopied in *cow* null mutants. Block of postsynaptic Wg signaling causes increased SV fusion frequency and amplitude of miniature excitatory junctional potentials (Speese et al., 2012). With neuronal *cow* RNAi, there is a similar increase in event frequency and amplitude. These results suggest a decrease in postsynaptic Wg signaling when *cow* is lost, supporting the Wg transport hypothesis. Blocking Wg secreted from neurons or glia increases muscle GluR cluster size, albeit with differential effects on neurotransmission efficacy (Kerr et al., 2014). Reducing neuronal Wg has no effect on mEJC frequency, but reducing glial-derived Wg increases SV fusion frequency (Kerr et al., 2014). Both nerve-evoked and spontaneous neurotransmission are increased in *cow* null mutants, together with increased Brp active zones and postsynaptic GluR clusters forming supernumerary synapses. SynapGCaMP is an exciting new tool to test function at individual synapses (Newman et al., 2017). With targeted neuronal *cow* RNAi, there is an increase in both the number of SV fusion events and the postsynaptic Ca^2+^ signal amplitude, which is consistent with both presynaptic and postsynaptic regulation of Wg signaling (Packard et al., 2002; Speese et al., 2012; Kerr et al., 2014). These functional phenotypes, combined with coordinated changes in presynaptic and postsynaptic formation suggest Cow regulates trans-synaptic Wg transport.

There were differences between spontaneous synaptic vesicle fusion findings between TEVC electrophysiological recordings and SynapGCaMP reporter (*MHC-CD8-GCaMP6f-Sh*) Ca^2+^ imaging (Newman et al., 2017). Motor neurons that presynaptically targeted *cow* RNAi showed stronger impacts on SV fusion frequency with imaging in contrast to recordings, comparable to effects in the *cow^GDP^* null mutants. Moreover, SynapGCaMP imaging revealed significantly larger SV fusion event magnitudes in contrast to the lack of change found with TEVC recording. While the basis of these differences in unknown, we speculate that it is due to the differential nature or sensitivity of these two methods. The Ca^2+^ imaging is based on measuring the change in the fluorescence signal over the baseline NMJ fluorescence (Δ*F*/*F*_0_; Newman et al., 2017), and it may be that glutamate receptor Ca^2+^ permeability or intracellular Ca^2+^ signaling dynamics is changed in a way not directly related to detectable membrane current changes in the *cow* mutants. TEVC recordings capture whole NMJ activity, whereas with imaging we only captured type 1b bouton activity normalized to area. In future studies, SynapGCaMP imaging can be used to map spatial changes in synapse function by assaying quantal activity separately in convergent type 1s and 1b motor neuron inputs and within discrete synaptic boutons (Newman et al., 2017). Moreover, differences between *cow^GDP^* and *cow^GDP^*/*Df* conditions could be influenced by second site-enhancing mutations on the Df chromosome. Overall, it should be noted that the changes in spontaneous SV fusion frequency and amplitude in *cow* mutants are subtle and variable, and need to be further studied in the future.

Wg is lipid modified via palmitoylation to become strongly membrane associated (Zhai et al., 2004). The hydrophobic moiety is located at the interface of Wg and Fz2 binding, shielded from the aqueous environment by multiple extracellular transporters until signaling interaction with the receptor (Takada et al., 2017). There have been many modes of extracellular Wg transport demonstrated, primarily from work in the wing disk, including microvesicles, lipoproteins, exosomes, and cytoneme membrane extensions (Greco et al., 2001; Panáková et al., 2005; Gross et al., 2012; Huang and Kornberg, 2015). These multiple mechanisms of transport are much less studied at the synapse; however, exosome-like vesicles containing the Wg-binding protein Evenness Interrupted (Evi) have been demonstrated at the *Drosophila* NMJ (Korkut et al., 2009). Cow could be considered an alternative extracellular Wg transport method (Chang and Sun, 2014), acting to shield Wg while facilitating transport through the extracellular synaptomatrix (Dani and Broadie, 2012; Dear et al., 2016). In addition, HSPGs have been shown to regulate ligands by stabilizing, degrading, or sequestering the ligand, or as bifunctional coreceptors, or as facilitators of transcytosis (Lin, 2004; Dani et al., 2012; Dear et al., 2017). Results presented here are consistent with the hypothesis that Cow is mediating Wg transport across the NMJ synapse (Chang and Sun, 2014), but also that Cow has an additional role in the negative regulation of Wg synaptic signaling.

The need for secreted Wg has been recently challenged, with Wg tethering to the membrane (*NRT-wg*) showing Wg secretion to be largely dispensable for development (Alexandre et al., 2014). In contrast, other recent studies suggest that Wg release and spreading is necessary (Beaven and Denholm, 2018; Pani and Goldstein, 2018; Stewart et al., 2019). We find tethering Wg at the NMJ synapse increases extracellular Wg ligand levels, with no change in mature bouton numbers. This Wg accumulation shows that *NRT-wg* is more stable at the synaptic signaling interface, consistent with other studies (Morata and Struhl, 2014; Chaudhary et al., 2019). However, although Wg levels increase, Wg signaling is less effective. With *NRT-wg*, only the budding of new satellite bouton is increased, with no increase in mature bouton formation. Reducing Wg function causes Fz2 upregulation (Cadigan et al., 1998; Chaudhary et al., 2019), so we hypothesize that Wg signaling could be maintained by increased presynaptic Fz2 receptors. When Wg is tethered, Cow cannot mediate intercellular transport, so the hypothesis predicts a similar phenotype with Cow (*NRT-wg*) or without Cow (*NRT-wg; cow^GDP^*). Indeed, Cow removal in the *NRT-wg* condition does not impact synaptic bouton number, although it does block the increase in satellite boutons, consistent with a Cow role in greater Wg stability (Chang and Sun, 2014). These results show that Wg secretion is required for the elevated NMJ development characterizing *cow* mutant animals.

To further test how Cow is working through the Wg pathway to negatively regulate NMJ development, we turned to genetic interaction tests with the Wg-negative regulator Notum (Gerlitz and Basler, 2002; Giráldez et al., 2002; Kakugawa et al., 2015). At the NMJ, Wg trans-synaptic signaling is elevated in the absence of Notum, and null *notum* mutants display larger NMJs with more synaptic boutons, increased synapse number and elevated neurotransmission (Kopke et al., 2017). All these defects are phenocopied by neuronal Wg OE, showing that the positive synaptogenic phenotypes arise from lack of Wg signaling inhibition. Consistently, genetically correcting Wg levels at the synapse in *notum* nulls alleviates synaptogenic phenotypes (Kopke et al., 2017). We show here that *cow* null mutants have the same phenotypes of expanded NMJs, supernumerary synaptic boutons, greater synapse number/function, and strengthened transmission, suggesting that Cow acts like Notum in regulating Wg signaling. We performed a genetic test to ask whether Cow and Notum work in this same pathway. While *cow* and *notum* null heterozygotes do not exhibit NMJ defects, *cow*/*notum* trans-heterozygotes display grossly expanded NMJs with excess boutons. This combined haplo-insufficiency (type 3 SSNC) of nonallelic noncomplementation suggests that Cow and Notum share related roles (Yook et al., 2001; Hawley and Gilliland, 2006). When we tested full double mutants, there is no additive effect, showing that Cow and Notum restrict Wg signaling in the same pathway. However, this pathway convergence appears restricted only to the control of structural synaptogenesis but not of functional neurotransmission, although the control neurotransmission amplitude was elevated in these studies.

Cow now joins the list of synaptic HSPGs with key roles in NMJ development. HSPGs have been implicated in vertebrate NMJ synapse formation for >3 decades (Kamimura and Maeda, 2017; Condomitti and de Wit, 2018). The Agrin HSPG is secreted from presynaptic terminals to maintain postsynaptic acetylcholine receptor clustering (Godfrey et al., 1984; Hubbard and Gnanasambandan, 2013). Another secreted HSPG, perlecan, regulates acetylcholinesterase localization (Peng et al., 1999; Arikawa-Hirasawa et al., 2002). *Drosophila* NMJ analyses have begun to more systematically elucidate HSPG roles in NMJ formation and function (Ren et al., 2009; Kamimura and Maeda, 2017). In particular, the glypican HSPG Dlp regulates Wg signaling to modulate both NMJ structure and function, including the regulation of active zone formation and SV release (Johnson et al., 2006; Dani et al., 2012; Friedman et al., 2013; Dear et al., 2017). Wg binds the core Dlp, with HS chains enhancing this binding, to retain Wg on the cell surface, where it can both compete with Fz2 receptors and facilitate Wg–Fz2 binding (Yan et al., 2009). This biphasic activity depends on the ratio of Wg, Fz2, and Dlp HSPG as expounded in the “exchange factor model” (Yan et al., 2009; Dear et al., 2016). Cow may impact this exchange factor mechanism as a fourth player, acting with Dlp to modulate Wg transport and Wg–Fz2 binding at the synaptic interface. It will be important to test Dlp levels and distribution in *cow* nulls to see how Cow fits into this model.

In addition to Cow, perlecan (Trol) is another secreted HSPG reported to regulate bidirectional Wg signaling at the *Drosophila* NMJ (Kamimura et al., 2013). Trol has been localized near the muscle membrane, where it promotes postsynaptic Wg accumulation. In the absence of Trol, Wg builds up presynaptically, causing excess satellite bouton formation (Kamimura et al., 2013). It is interesting to note that *cow* mutants enhance Wg signaling without increasing satellite boutons. In *trol* mutants, ghost boutons increase due to decreased postsynaptic Wg signaling (Kamimura et al., 2013). Note that cow mutants do not exhibit ghost boutons, which fails to support decreased postsynaptic Wg signaling. Other postsynaptic defects in *trol* mutants (e.g., reduced SSR, increased postsynaptic pockets; Kamimura et al., 2013) are NMJ ultrastructural features that could be a future focus using electron microscopy studies. Similar to *cow* mutants, extracellular Wg levels are decreased in the absence of Trol, speculated due to increased Wg proteolysis, since HS protects HS-binding proteins from degradation (Saksela et al., 1988). In *cow* mutants, it is not yet known whether Wg is decreased due to elevated signaling (ligand/receptor endocytosis) or to increased degradation due to Cow no longer protecting/stabilizing the ligand. Given that synaptic Fz2 is internalized with Wg binding (Mathew et al., 2005), future experiments could test internalized Fz2 levels in *cow* mutants as a proxy of Wg signaling.

In summary, we have confirmed here new tools to study Cow HSPG function, and have discovered that Cow from presynaptic motor neurons restricts NMJ bouton formation, glutamatergic synapse number, and NMJ functional differentiation. Cow acts within the same Wg trans-synaptic signaling pathway as Notum by regulating the Wg ligand in the extracellular synaptomatrix. Secreted Cow modulates extracellular Wg ligand levels, with additional functions controlling Wg signaling efficacy, which may be independent of or dependent on Wg transport. It will be interesting to determine whether Cow core protein and/or its HS chains are important for the synaptic structural and functional phenotypes. Wg must be secreted for Cow to act on it, as shown by the membrane-tethered interaction studies, showing that secreted Cow must work on the freely diffusible Wg ligand. Perhaps most informative for our future studies will be dissection of the interactions, coordination or redundancy of the multiple synaptic HSPGs at the NMJ, to further the understanding of extracellular Wg trans*-*synaptic signaling regulation during synaptic development. *Drosophila* is a particularly well suited model to study HSPGs because of the relatively reduced complexity in this system (17 HSPGs in mammals vs 5 HSPGs in *Drosophila*; Sarrazin et al., 2011). We look forward to expanding future studies to examine multiple synaptic HSPGs in parallel, with the goal of elucidating the surprisingly complex control of trans-synaptic signaling occurring within the extracellular synaptomatrix.

## Synthesis

Reviewing Editor: Lorna Role, NINDS Decisions are customarily a result of the Reviewing Editor and the peer reviewers coming together and discussing their recommendations until a consensus is reached. When revisions are invited, a fact-based synthesis statement explaining their decision and outlining what is needed to prepare a revision will be listed below. The following reviewer(s) agreed to reveal their identity: Quan Yuan.

SUMMARY

The authors of this manuscript examined functions of Carrier of wingless (Cow), a secreted Drosophila HSPG, in limiting synaptic bouton formation and neurotransmission at the glutamatergic NMJ, potentially through suppressing the Wnt signaling. To analyze the phenotypes associated with either reducing or elevating the levels of Cow, the authors quantified several key parameters related to the structural and functional organizations of the NMJ synapses. These include the anatomical assessment of the total bouton number, the percentage of satellite boutons, the measurements on synaptic transmission and vesicle fusion by electrophysiology recordings and functional imaging, and lastly, the quantifications of the number of presynaptic active zones and the postsynaptic GluR clusters by high resolution SIM microscopy. In addition, the authors performed genetic interaction experiments to explore the role of Cow as the carrier or suppressor of extracellular Wg signaling.

Overall, the manuscript is clearly written with a good amount of background information. The experiments described in the manuscripts were well justified, properly controlled and quantified. The evidence supporting Cow as a new synaptic HSPGs with key roles in NMJ development is convincing.

However, due to the complexity of the molecular pathways regulating the Wg signaling and the relatively mild phenotypes generated by the genetic manipulations, the functions of Cow as a Wg regulator necessitates further clarifications.

MAJOR ISSUES:

The four major issues that must be addressed, in order of their importance, are as follows:

1. Additional data or clarifications are needed to support the genetic interaction

The double heterozygous null condition cow[GDP]/notum[KO] results are over-interpreted. For Lines, 503-504, the Yook et al. 2001 paper is cited. However, that paper does not state that double het null interactions are evidence for an in vivo interaction; it explicitly suggests something else. That paper suggests that double het conditions may be indicative of a physical interaction (or an interaction through a complex) if at least one mutation renders a partially functional or poisonous gene product. The non-complementation observed in the present study is consistent with both Cow and Notum both acting to restrict Wg signaling - but to say “the same pathway” might be a stretch.

To definitively conclude a single pathway, it would be more convincing to have a full null condition in at least one of the genes and to show that loss of the second gene does not enhance the null phenotype of the first. As the data stand, it appears like there might be a little additivity going on (Fig 7A) because the cow[GDP]/+ condition does have a numerically elevated bouton number (Line 516 says “indistinguishable” but the stats say p = 0.09).

In terms of required revisions, the authors could pursue the double mutant experiment suggested (with at least one condition being a full null) or they could soften the interpretation of what the non-complementation actually means

2. Issues with the Physiology experiments:

• The authors recorded from three genotypes: w[1118], cow[GDP] homozygotes, and cow[GDP]/Df. However, the only statistical comparisons are between w[1118] and cow[GDP]/Df. It is not clear why the authors excluded the GDP homozygote from the statistical analysis. If the GDP allele is null, and if the physiology phenotypes are strictly due to cow loss, then the GDP homozygous phenotypes should be just as strong as the GDP/Df phenotypes.

• Second, it is not clear why the vglut > RNAi physiology analysis is done in this separate way (with GCaMP). The MHC-CD8-GCaMP6f-Sh line used by the authors is an excellent tool for garnering spatial release information at an NMJ, but a spatial analysis is not what is being done. The text indicates that it “confirms” increased frequency (Line 413), but the best way to confirm increased frequency is simply to conduct electrophysiological measurements in the vglut > RNAi condition; electrophysiology is more sensitive than the GCaMP reagent.

• The GCaMP measurements suggest a greater quantal amplitude for vglut > RNAi. That is not a phenotype shared by the null conditions, so it is not clear if this is due to the changed genotype or the changed technique.

• Finally, if Notum is acting in the same process as Cow, then the double hets in Figure 7 should also phenocopy the cow mutants for electrophysiology phenotypes.

3. information on the expression pattern of Cow protein in neuronal and non-neuronal tissues.

The expression and localization of Cow is central to the conclusion of the study. There are multiple incidences in the manuscript, where the authors mentioned possible non-neuronal functions of Cow (Fig. 2C and Discussion Line 600-601). However, the author did not provide an overall evaluation of the Cow protein distribution in the larval CNS and NMJ. Since the antibody has been produced and tested in this study, the author should perform immunohistochemistry study and provide the information, with the Cow null as the control.

4. full disclosure of the statistics and comparisons.

The authors need to address overall issues with the statistical analyses and reporting. A part of the data is currently hidden in the table. The results of statistical analyses and their implications should be included in the results section in the revised manuscript

At many places in the text and figures, the authors appear to highlight favorable statistical comparisons, while not discussing other equally important comparisons. Those additional comparisons were calculated, and they can be read on the Table (p. 58 of the PDF), but they do not appear in the text or figures. The solution for this would be to go through the manuscript figure-by-figure and to report all comparisons with actual p values (not simply *** for two chosen genotypes or “n.s.” for others) - and also to report all relevant comparisons in the text.

Here are some cases where statistics or data reporting is consequential for interpretation:

• Figure 3: The text alludes to a 24B > UAS-cow experiment being done (Lines 371-378), but there are no images or numbers to correlate with the text.

• Figure 4B-D: The cow[GDP]/cow[GDP] homozygous condition has marginally increased EJC amplitude, but this increase does not achieve statistical significance (p = 0.09). Moreover, the homozygous [GDP] condition seems to have no statistically significant increase in mEJC frequency (p = 0.57; contrast with text in Lines 407-408). If this is a null condition, and if the phenotypes are due to cow loss alone, then the homozygous GDP line should be as severe as cow[GDP]/Df. The authors need to explain these discrepancies.

• Figure 4E-F: Low n makes these comparisons difficult, but the frequency number (marked as *) actually has p = 0.0513, which most people would say does not quite achieve statistical significance.

• Figure 6B: In the FRT-wg background the cow[GDP] condition increases bouton number (p = 0.03), and in the NRT-wg background, the cow[GDP] condition does not increase bouton number (p = 0.99). The “n.s.” highlighted here between the first and last columns does not make much sense.

• Figure 6C: In the NRT-wg genetic background, the cow[GDP] condition significantly decreases satellite bouton number (p = 0.0038). That is an interesting finding.

• Figure 7A: The double het is also statistically significantly different than the single hets.

ADDITIONAL POINTS:

1. The cow[5Delta] allele is intriguing. This reviewer does not disagree with the decision to exclude it after Figure 1. However, do the authors have any phenotypic data from third-instar survivors with this allele? Did they show some of these same phenotypes? Is it lethal over deficiency?

2. An explanation of the satellite bouton phenotype does not appear until lines 590-592 in the discussion, and for most readers it would probably be helpful to have earlier, so the satellite bouton quantifications do not seem like an add-on phenotypic analysis.

3. Fig. 2B, the quantification of the percentage of satellite boutons should be provided. The author should also provide an explanation on the similar increases of satellite boutons observed in Cow knock-down (Fig. 2B) and Cow overexpression (Fig. 3D).

4. Figure 5: The increased Brp punctae number and decreased volume are intriguing results and consistent with other reports that show a reciprocal relationship between AZ number and Brp volume (e.g., the rab3[rup] mutant shows this phenotype strikingly).

Is total Brp protein/unit synapse area maintained (or could the authors report this number from the measurements they have already taken? It is hard to know what the puncta number value means for physiology, when those increased number corresponds to decreased volume. The authors state that the increased Brp number correlates with the increased spontaneous frequency, but this reviewer is not quite that would be true.

5. Stylistic: In the results section the authors often repeat the exact same fact twice in a row, the second time with quantification. See Lines 345-347 for one example or 348-355 for another.

## Author Response

We submit here our revised study for your 90-day deadline. The overall consensus was that “the manuscript is clearly written with a good amount of background information. The experiments described in the manuscript are well justified, properly controlled and quantified. The evidence supporting Cow as a new synaptic HSPGs with key roles in NMJ development is convincing.” This revision contains 1 entirely new figure (Figure 2), a figure with new data (Figure 8), and newly added p-values in all figures. Text changes in the revised manuscript are marked throughout with yellow highlight. Below, we respond point-by-point to your compiled review.

Major Points

There were four points to be addressed, listed in order of priority:

1. Additional data or clarification to support the genetic interaction of the double heterozygous null condition cow[GDP]/notum[KO]. The non-complementation is consistent with both Cow and Notum both acting to restrict Wg signaling, but to say “the same pathway” might be a stretch.

To definitively conclude a single pathway, it would be more convincing to have a full null condition in at least one of the genes and to show that loss of the second gene does not enhance the null phenotype of the first, or soften the interpretation of non-complementation.

As suggested, we performed the proposed experiment. We used the full double mutant as the strongest test of non-complementation. We found that the cow[GDP]/notum[KO] double null mutant does not have a stronger phenotype than either single null mutant alone, demonstrating that Cow and Notum act to restrict synaptic Wg signaling in the same pathway, as suggested by the reviewers. These new results have been added to the text and to revised Figure 8.

2. Issues with the physiology experiments:

• The authors recorded from 3 genotypes: w[1118], cow[GDP] homozygotes, and cow[GDP]/Df. However, the only statistical comparisons are between w[1118] and cow[GDP]/Df.

We performed further recordings on cow[GDP] homozygotes and show statistical comparisons for all 3 genotypes. New data and statistical comparisons are shown in revised Figure 5.

• Second, it is not clear why the vglut>RNAi analysis is done with GCaMP.

MHC-CD8-GCaMP6f-Sh measurements show an increase in spontaneous fusion frequency and amplitude. TEVC recordings show the same trends, but results are not statistically significant. We show the results of both assays in the revised manuscript and discuss the differences.

• Finally, if Notum is acting in the same process as Cow, then the double heterozygotes should also phenocopy the cow mutants for electrophysiology phenotypes.

As suggested, we did TEVC recordings on the cow[GDP]/notum[KO] double heterozygotes, but did not detect any change in synaptic function. This may reflect a differential sensitivity of the genetic interaction test for synaptic function versus structure, or a real difference in the control mechanisms for synaptic function versus structure. New results are discussed in the revision.

3. Information on the expression pattern of Cow protein in neuronal and non-neuronal tissues.

As suggested, we provide more information on Cow protein expression in both neuronal and non-neuronal tissues in a dedicated new figure (Figure 2). This includes 1) anti-Cow antibody labeling in embryos, NMJs and wing discs, as well as 2) Cow-Gal4 driving UAS-Cow::eGFP.

4. Full disclosure of statistical comparisons, reporting all comparisons in the figures and results with p values (not using not significant (n.s) and asterisk (*) comparisons in figures).

As directed, we have replaced all asterisks and not significant (n.s) in figures with the p values. We similarly report all text results with p values throughout the revised text. We do not report every possible comparison in the text because it is extremely hard to read (all in Stats Table).

Additional Points

1. The cow[5Delta] allele is intriguing. This reviewer does not disagree with the decision to exclude it after Figure 1. However, do the authors have any phenotypic data?

We are happy the reviewer agrees with our decision. Chang and Sun, 2014 report cow[5Delta] to be a genetic and protein null, albeit with normal RNA expression. We show this allele is not a genetic or protein null, and harbors second site lethality. Cow loss permits normal adult viability. For these reasons, we characterize a true null allele (cow[GDP]) and don't include cow[5Delta].

2. An explanation of the satellite bouton phenotype does not appear until lines 590-592 in the discussion, and for most readers it would probably be helpful to have earlier.

As suggested, we now mention the satellite bouton phenotype earlier within the revised text.

3. The authors should provide an explanation on the similar increases of satellite boutons observed in Cow knockdown (Fig. 2B) and Cow overexpression (Fig. 3D).

As suggested, we draw attention to this observation and discuss it further in the revision.

4. Figure 5: The increased Brp punctae number and decreased volume are intriguing results and consistent with other reports that show a reciprocal relationship.

As indicated, we add another reference highlighting this relationship. We find total Brp/unit synapse area is maintained, and we do not know how the reduced Brp punctae volume would affect synaptic physiological function. Therefore, as suggested, we have deleted the suggestion that the increased Brp punctae correlate with the increased spontaneous SV fusion frequency.

5. Stylistic: In the results section the authors often repeat the same information twice in a row, the second time with quantification. See Lines 345-347 for one example or 348-355 for another.

As suggested, this is a stylistic choice. We present qualitative results followed by quantitative results. We have kept this style consistent throughout the revised manuscript.
